# A Global Perspective on Sustainable Show Cave Tourism

**DOI:** 10.1007/s12371-022-00717-5

**Published:** 2022-06-28

**Authors:** Veronica Chiarini, Jochen Duckeck, Jo De Waele

**Affiliations:** 1 Department of Biological, Geological and Environmental Sciences, University of Bologna, Via Zamboni 67, 40126 Bologna, Italy; 2 Nuremberg, Germany

**Keywords:** Geotourism, Karst, Sustainable management, Nature protection, Cave monitoring, Show caves

## Abstract

Caves are among the most visited geological features in the world, attracting over 70 million people every year in more than 1,200 caves worldwide, and amounting up to 800 million Euros in entrance fees alone. The global business of show caves employs roughly 25,000 people directly (management, guides), and at least 100 times more people if we consider the connected tourist activities (souvenir shops, local transport, travel agencies, restaurants, and bars). It is estimated that the whole show cave business has a global commercial value of roughly 2 billion Euros, a number that is increasing constantly. Show caves are generally fragile ecosystems, and care should be taken in their management to safeguard their value for future generations. The international scientific (speleological) community has issued international guidelines for the sustainable development and management of show caves eight years ago, but their application is still far from being applied globally, especially in developing and least developed countries. Cave tourism is expected to increase, especially in countries where caves are abundant but not yet considered as tourist attractions, and where economic and political instability slow down the development of tourism. There are still a lot of possibilities for the opening of new show caves, especially in countries with low Gross Domestic Income (GDI), but their management needs to be sustainable, so that caves become a means of sustaining local economies, educating people on these fragile geo- and ecosystems, and protecting contemporarily their scientific and cultural heritage for future generations.

**Supplementary Information:**

The online version contains supplementary material available at 10.1007/s12371-022-00717-5.

## Introduction

Caves have been used by our ancestors since prehistoric times, often limiting frequentation to the areas close to the entrance, where daylight still penetrates, and the fireplace smoke could easily be dispersed without suffocating these early sheltering inhabitants. From at least 64,000 years ago Neanderthal people first, Modern Humans later, have started going deeper into caves, as testified by their rudimental (La Pasiega Cave, Cantabria, Hoffmann et al. 2018) and story-telling rock art (Sulawesi, 43.9 ka, Aubert et al. 2019). These deeper explorations were often driven by cultural needs (worshipping) or later also by the need of exploiting local resources such as flint or precious salt minerals (e.g. mirabilite or gypsum). Later on, caves were visited for several reasons, but records are often very fragmentary, such as the one of Assyrian King Shalmaneser III to the springs and caves of the Tigris River around 853–852 B.C. (Shaw 1992). The oldest historical inscriptions in caves are those found in Lu Di Yan (Reed Flute Cave) dating back to 792 A.C. (Tang Dynasty), but it is not known whether these visits were guided or whether they were just occasional and self-guided tours by some early adventurers. Postojna Cave, in Slovenia, has old signatures dating back to the dark Medieval times (a signature reporting “C.M.” and the probable date of 1213) (Kempe and Hubrich 2011).

According to the International Show Cave Association (ISCA) a show cave is defined as “a natural occurring void beneath the surface of the earth that has been made accessible to the public for tours” (Cigna 2019). Many caves in the world would fall under this broad definition. Most show caves have three things in common: 1) you need to pay an admission fee; 2) the cave has some kind of infrastructure that facilitates access (pathways, stairs, lights, artificial entrance, etc.); 3) visits are generally carried out under the supervision of a guide.

**Fig. 1:**
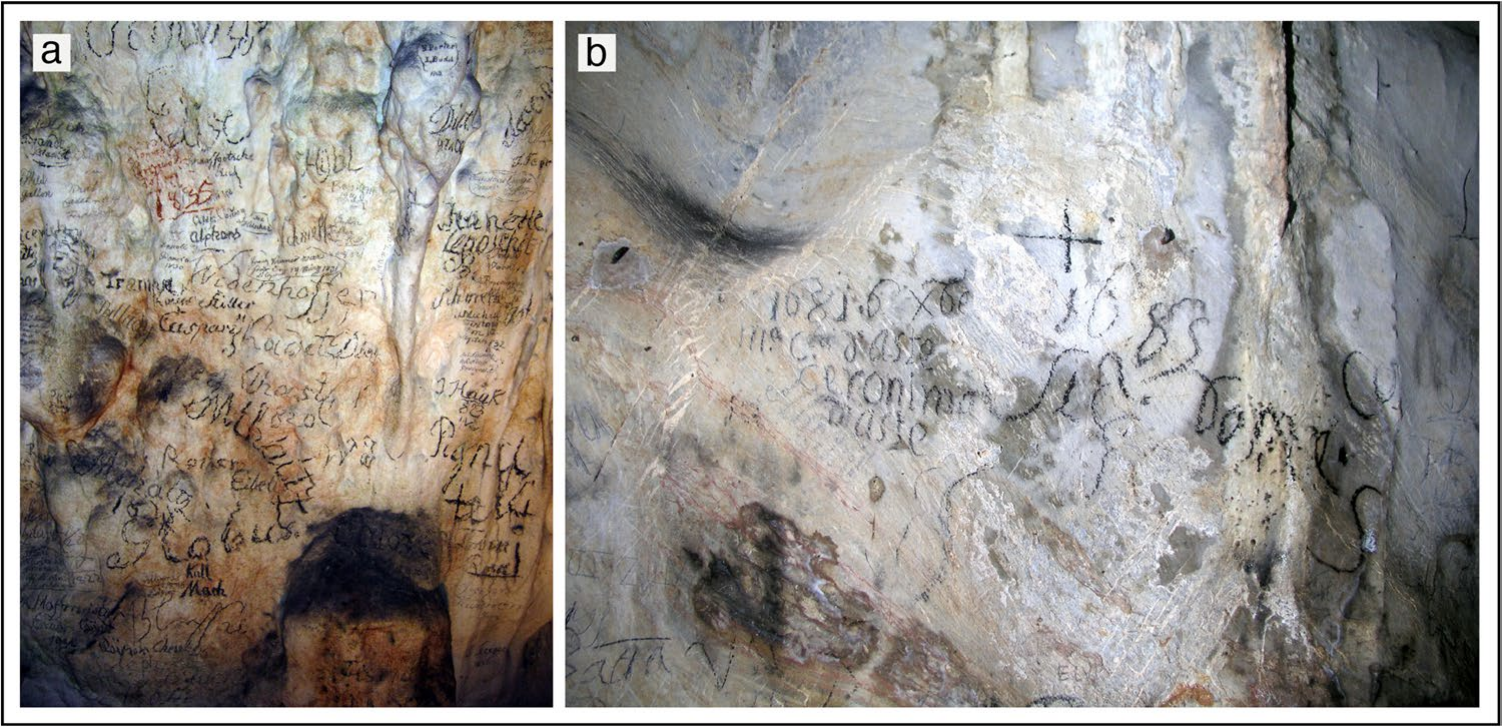
Signatures of visitors in (**a**) the “Speleovivarium” of Postojna Cave, Slovenia, where visitors left their signs at least since the sixteenth century, and maybe even earlier (photograph by Jo De Waele); (**b**) seventeenth-century signatures in the Grotta di Santa Lucia superiore, Toirano (northern Italy), massively visited by pilgrims since the fifteenth century (photograph by Jean-Yves Bigot)

Guided visits to some of the oldest known show caves were surely taking place since the thirteenth century and became rather common especially in the fifteenth and sixteenth century, as shown by signatures on walls in Jasovská Cave in Slovakia (inscriptions from 1452) (Hvizdák et al. 2014), Sontheimer Höhlen in Germany with repeated visits by Herzog Ulrich von Württemberg (1516) (Shaw 1992), and Postojna Cave, where sixteenth-century signatures are frequently seen (Fig. 1) (Kempe et al. 2006; Kempe and Hubrich 2011). Some kind of adaptation was often carried out in these early visited caves, to make the paths somewhat more comfortable for people not used to enter dark and damp places such as caves.

**Fig. 2:**
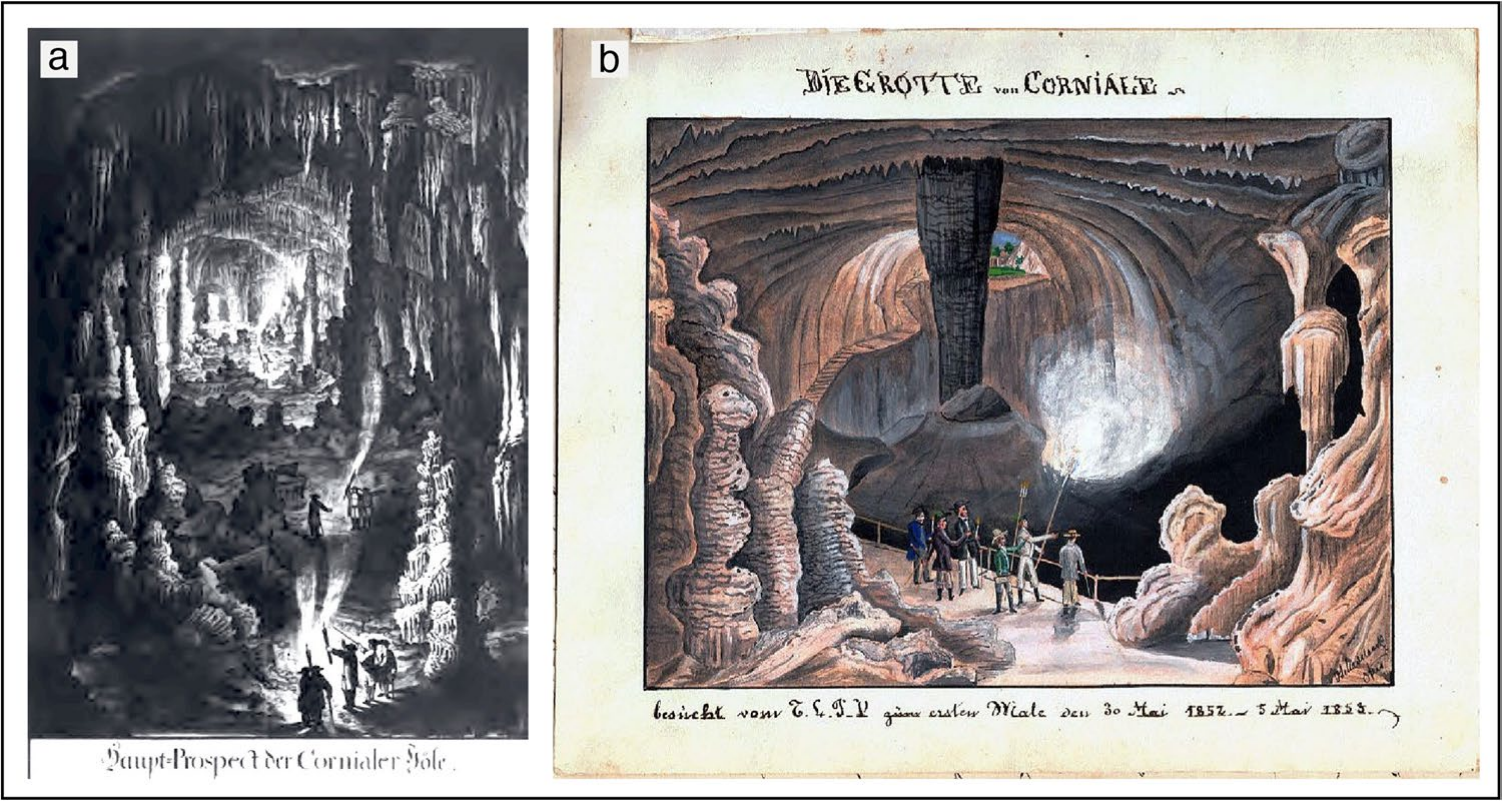
A representation of the large halls in Vilenica cave (also known as Corniale Cave), close to Sežana, Slovenia. (**a**) By Nagel (1784) and (**b**) by an anonymous traveller in a manuscript entitled “Das buch unserer ausflüge durch berg & thal.” (Trieste, 1852) (courtesy of Library “Centro di Documentazione Speleologica F. Anelli”, Bologna)

Most probably, some kind of payment was asked for to enter the cave since the early beginning of underground visits, but these “fees” were probably extemporary. It appears logical to hypothesise that occasional paid visits might have occurred since Roman times, although a solid documentation on this has not been found in the literature. For example, some cave entrances with exiting hot air of the Kronio cave complex, near Sciacca (Sicily, Italy), were used as a *calidarium* since Greek and especially Roman times (Badino and Torelli 2014). If we define a show cave as one in which an admission fee needs to be paid on a regular basis, then Vilenica (Corniale, in Italian), close to Sežana in Slovenia, can be considered the first show cave in the world (Fig. 2) (Cigna and Forti 2013). The first reported payment of a ticket to enter this cave dates back to 1633, when Count Benvenut Petac started charging cave visitors, donating the money to the local church of Lokev (Cigna 2019). During this early-stage tourism phase, caves were often visited with local guides: groups were usually composed of few adventurous visitors relying on the experience of these early cave guides. The first official cave guide appears to be Valentin Wagner, working on stable terms from 1649 in Baumannshöhle, in the Harz Mountains in Germany (Kempe et al. 2004).

**Fig. 3:**
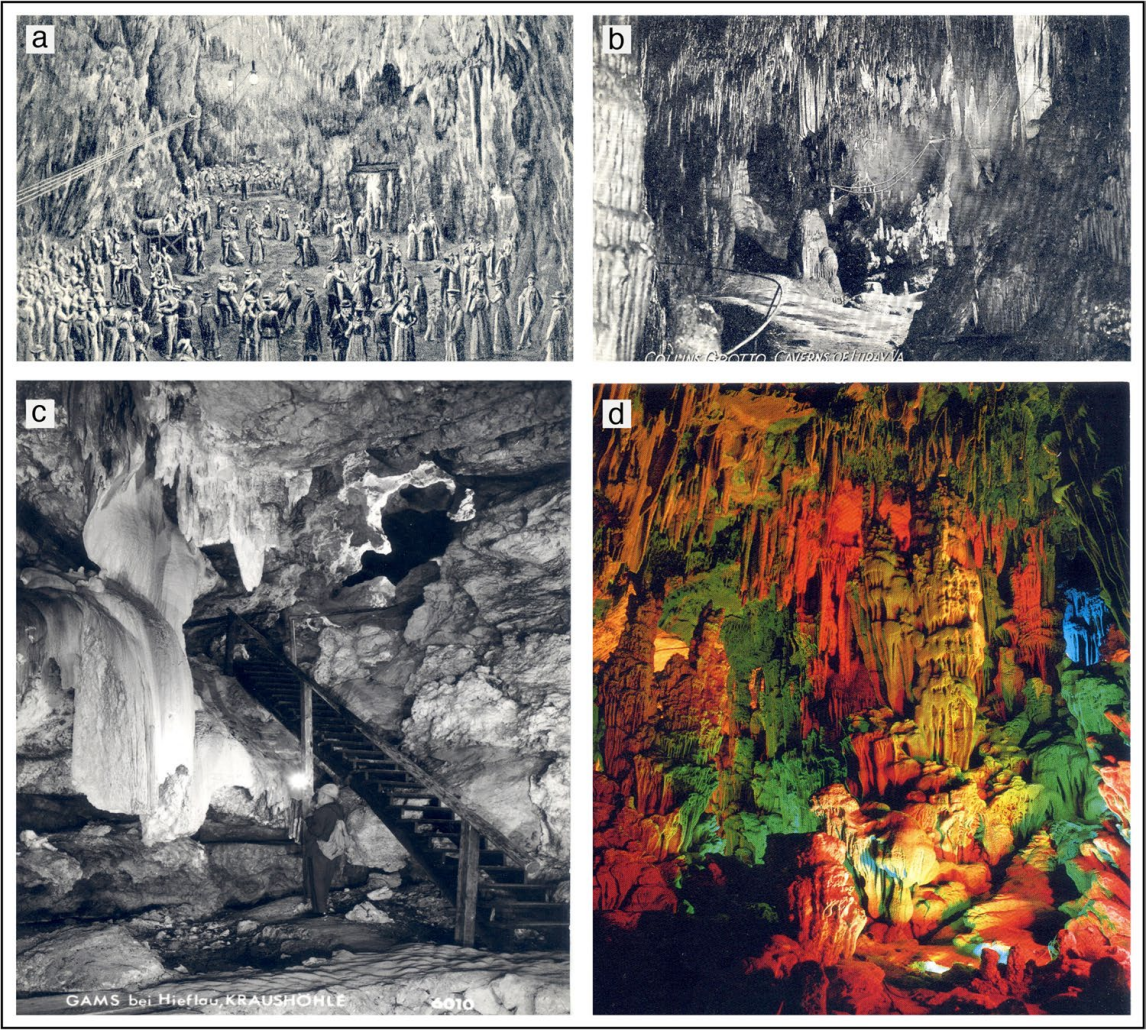
Some old drawings and postcards of historical show caves: (**a**) a Postojna postcard of 1900, showing the ballroom; (**b**) a 1906 postcard of Luray Caverns (photograph by J.D. Strickler); (**c**) a 1980 postcard of Kraushöhle, in Austria with historical visit using carbide lights; (**d**) the typical colourful scenery of a Chinese cave (Lu Di Yan or Reed Flute Cave), where caves are mostly used as “sceneries” and not as scientifically interesting “world-aparts”. Postcards are courtesy of library “Centro di Documentazione Speleologica F. Anelli”, Bologna

At the beginning, different lighting systems were used to illuminate these dark environments, including candles, torches, magnesium wire, oil lamps, and gas, but it was the introduction of electric lights that provided an important impulse to the development of cave tourism (Shaw 2003). Indeed, this promising new technology was experimented in caves only one year after the patenting by Thomas Edison of the vacuum light bulb with carbon filament. It was the 22^nd^ of July 1880 when in Chifley Cave, which is part of the famous Jenolan Caves in New South Wales (Australia), Lieutenant Colonel E.C. Cracknell introduced for the first time an electrical illumination in a show cave (Cook 1889). A rudimental infrastructure was built in the following years, and part of Jenolan Cave was paved to allow the installation of a wiring system empowered by a heavy battery. This work was completed in 1887 (Betteridge 2019). More permanent installations were made in Sloupsko-Šošùvské Jeskyně (Czech Republic, 26 July 1881) and Luray Caverns (Virginia, USA, September 1881) (Shaw 2003), where electric arc lamps with carbon electrodes were used (Fig. 3b). In 1883 Kraushöhle (Austria) was the first to use the light bulbs similar to those invented by Edison, although they functioned only for 7 years, and were then substituted by carbide lamps (Fig. 3c) (Shaw 2003). Postojna Cave, the largest and most visited show cave in Europe, installed its electric lights in 1884 (Fig. 3a) (Shaw 2003).

In the twentieth century many more caves started to be used for tourist purposes, creating local income to private owners, local communities, caving organizations, private enterprises, and companies. Caves are indeed among the most important geotourism resources in the world, attracting large amounts of visitors (Cigna and Forti 2013). Although none of the show caves reaches the enormous numbers of tourists visiting famous geological landmarks such as Grand Canyon (5.9 million visitors per year), Yosemite N.P. (4.5 million/year), Yellowstone N.P. (4 million/year), or the Shilin Stone Forest in China (4 million/year), some of them (e.g. Huanglong Dong (Yellow Dragon Cave) and Lu Di Yan (Reed Flute Cave)) have over 1 million visitors/year (Fig. 3d). Earlier estimates of the importance of show caves reported around 500 caves with over 50,000 tourists/year, and 150 million visitors globally, worth around 3.5 billion US $ (c.a. 2 billion Euros) (Cigna 2016, 2019). A dedicated website (www.showcaves.com, consulted in 2021) lists almost 1400 show caves, but they include artificial mines, caves not open to public anymore, and duplicate entries (show caves with two names).

Many papers have been published dealing with show caves and their management in the world (Cigna et al. 2000; Cigna and Burri 2000; Cigna and Forti 2013; Spate and Spate 2013; Cigna 2019), and in different countries, including France (Biot 2006), Australia and China (Crane and Fletcher 2016), China (Cao et al. 2017), Brazil (Lobo et al. 2008; Lobo and Moretti 2009), Croatia (Bočić et al. 2006), Italy (Garofano and Govoni 2012), Romania (Meleg et al. 2019), Serbia (Tomić et al. 2019), Slovenia (Tičar et al. 2018), and the USA (Foster 1999). The worldwide increasing interest of people in visiting show caves has led to the development of this kind of tourism in several countries, also becoming an important economic income, especially for local communities in the proximities of these tourist sites. However, opening of caves to mass tourism has caused an increasing pressure on the vulnerable karst and cave environment, giving rise to global concerns on the management and protection of these important geo-ecosystems (Watson et al. 1997, Gillieson et al. 2022). A sort of Environmental Impact Assessment (EIA) was proposed at the start of the twenty-first century (Cigna and Burri 2000; Cigna et al. 2000). In these last 20 years, the caving associations in developed countries have increasingly been involved in the preliminary assessment of future show caves, since this process requires a combination of different expertise, including marketing (market surveys: e.g. will the show cave be attractive to visitors, and thus be economically sustainable?), economy (economic constraint: e.g. how much money is needed to equip the caves, and the related infrastructure and accessibility?), and cave and karst environment knowledge (to define critical factors of geological, biological, and environmental nature through monitoring campaigns and scientific investigations). At present, this approach is not (or is poorly) adopted in developing countries. In such countries, cavers, if present, are poorly organised and the scientific karst community is often not well established or has poor influence in the political and socio-economic spheres, with greater consequences on the sustainability (both economic and environmental) of show cave tourism.

This paper aims at giving a general overview on show caves in the world, providing information about their location and geographical distribution and some statistics on the economy generated by them based on pre-COVID data available online, providing a fairly detailed picture on this profitable business in various parts of the world. It is in fact certain that the number of show caves will further increase in the next decades, especially in developing or least developed countries. This paper also provides some general guidelines, based on well-established international practices, that will allow future developments of show caves to be foresighted and sustainable, making the use of these precious karst resources both profitable and endurable.

## Caves, fragile geo-ecosystems

Caves can be considered natural voids in the Earth’s crust, and in an anthropocentric perspective they should be big enough to be potentially explored by humans (Ford and Williams 2007, p. 209).

Their formation depends on several geological processes which are used as a reference for cave classification: wind, volcanism, tectonics, ice, and dissolution in water, which is the major process involved in speleogenesis. As a result, the majority of Earth’s cave systems are mainly represented by carbonate rocks, even if remarkable karst phenomena occur in gypsum, halite, and poorly soluble rocks such as quartzites (Wray and Sauro 2017). A significant number of caves are also hosted in lava fields, with classical lava tubes being the most representative (Sauro et al. 2020).

**Fig. 4:**
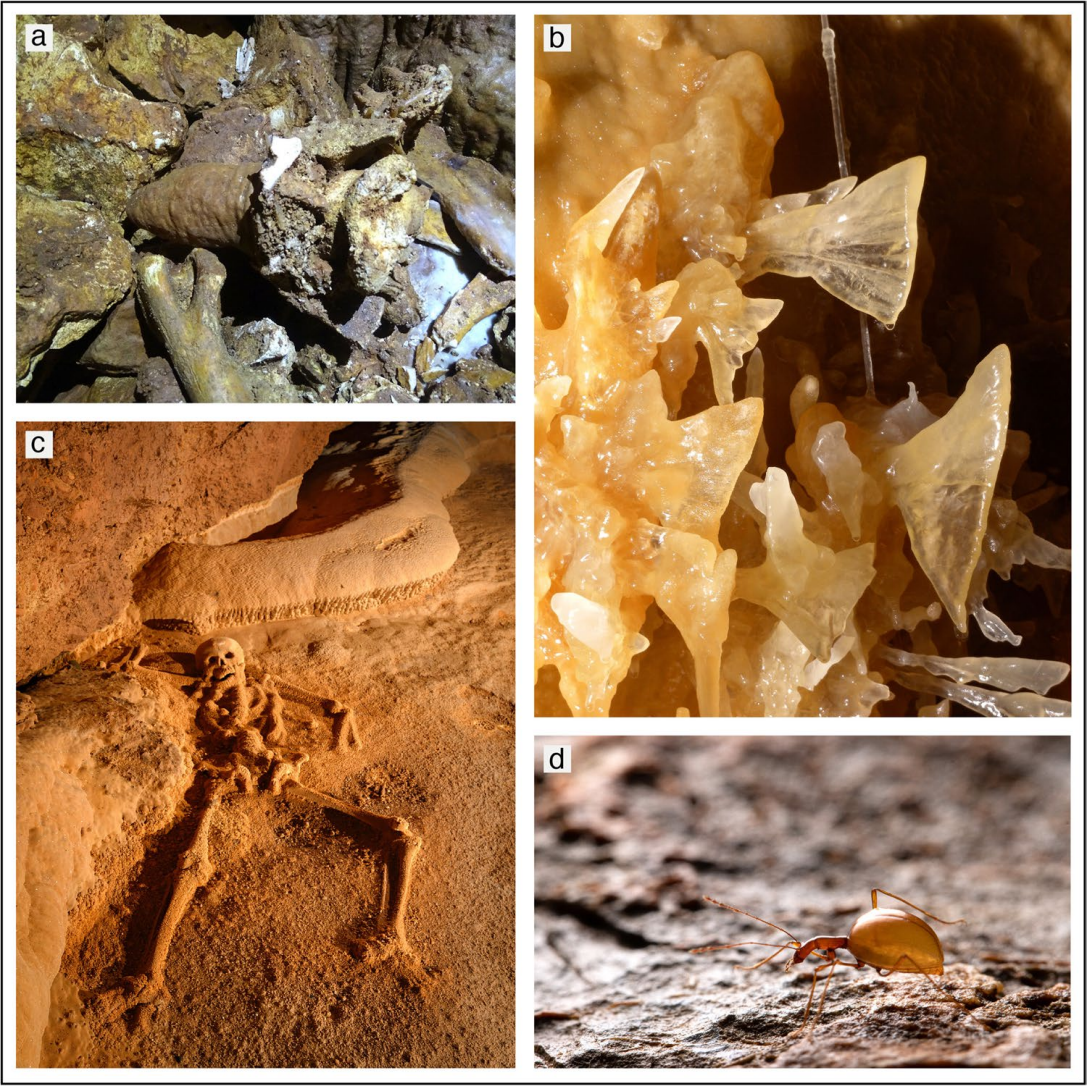
(**a**) Stalagmite growing on vertebrate bones in Pozzo Cucù cave, Apulia (southern Italy). The stalagmite is > 110 ky old, so the bones are of extinct Pleistocene animals (photograph by Jo De Waele); (**b**) the famous delicate “butterfly” helictites in Sonora Caverns, Texas (USA), the most famous of which was damaged by a visitor in 2006 (photograph by Lukas Plan); (**c**) the Crystal Maiden, a Maya skeleton of a young girl, died over one thousand years ago, in Actun Tunichil Muknal Cave, Mexico (photograph by Mark Burkey); (**d**) *Leptodirus hochenwartii*, the first troglobitic species described from Slovenian caves (photograph by Teo Delic)

Caves are unique geo-ecosystems thanks to their strong geodiversity, their relatively stable environmental conditions, the absence of strong seasonal modifications, and permanent darkness. They can also be considered “conservative environments” able to preserve information for long period of times. As an example, information about past environmental and climate oscillations can be preserved in cave sediments (e.g. pollen content) and speleothems (Fig. 4a) (e.g. calcite stable isotope composition, petrography, trace elements, etc.) (Fairchild and Baker 2012 and references therein). Their specific environmental conditions can lead not only to the precipitation of rare speleothems (Fig. 4b) and mineral phases, making these environments extremely interesting for earth science research (Hill and Forti 1997), but also to the creation of a specific habitat which is extremely interesting for the study of ecological adaptation of both vertebrates, invertebrate species (Mammola 2019; Mammola et al. 2020) (Fig. 4d) and bacterial colonies (Barton and Northup 2007), with a potential important impact for medical research and for the understanding of the origin of life on Earth (Barton and Northup 2007). Given the characteristics of stability and the “conservation” properties of these environments, caves can also be a source of information about past cultures and the origin and evolution of the genus *Homo,* since they were often used as shelters or for ritual purposes (Fig. 4c) (e.g. Facorellis et al. 2001; Sadier et al. 2012; Herries et al. 2020). Thus, also archaeologists and anthropologists find these places particularly interesting for the huge amount of information they can provide.

However, the same geological and environmental characteristics which provide caves with an extremely important scientific value make them vulnerable environments, which can be easily damaged causing an irreparable loss of scientific information and natural habitat. These environments are particularly sensitive to the activities carried out at the surface above and in the recharge area of the karst systems (Gillieson 2011). Indeed, caves are a fundamental part of the subterranean drainage network, whose recharge lies in the surrounding catchment areas. Not only water, but also air masses are exchanged between the external atmosphere and the internal cave environment (Badino 2010). These fluxes of water and air are the main natural perturbators of the subterranean world, causing subtle changes in air temperature, chemical composition of waters and air, and physical movements of fluids.

**Fig. 5:**
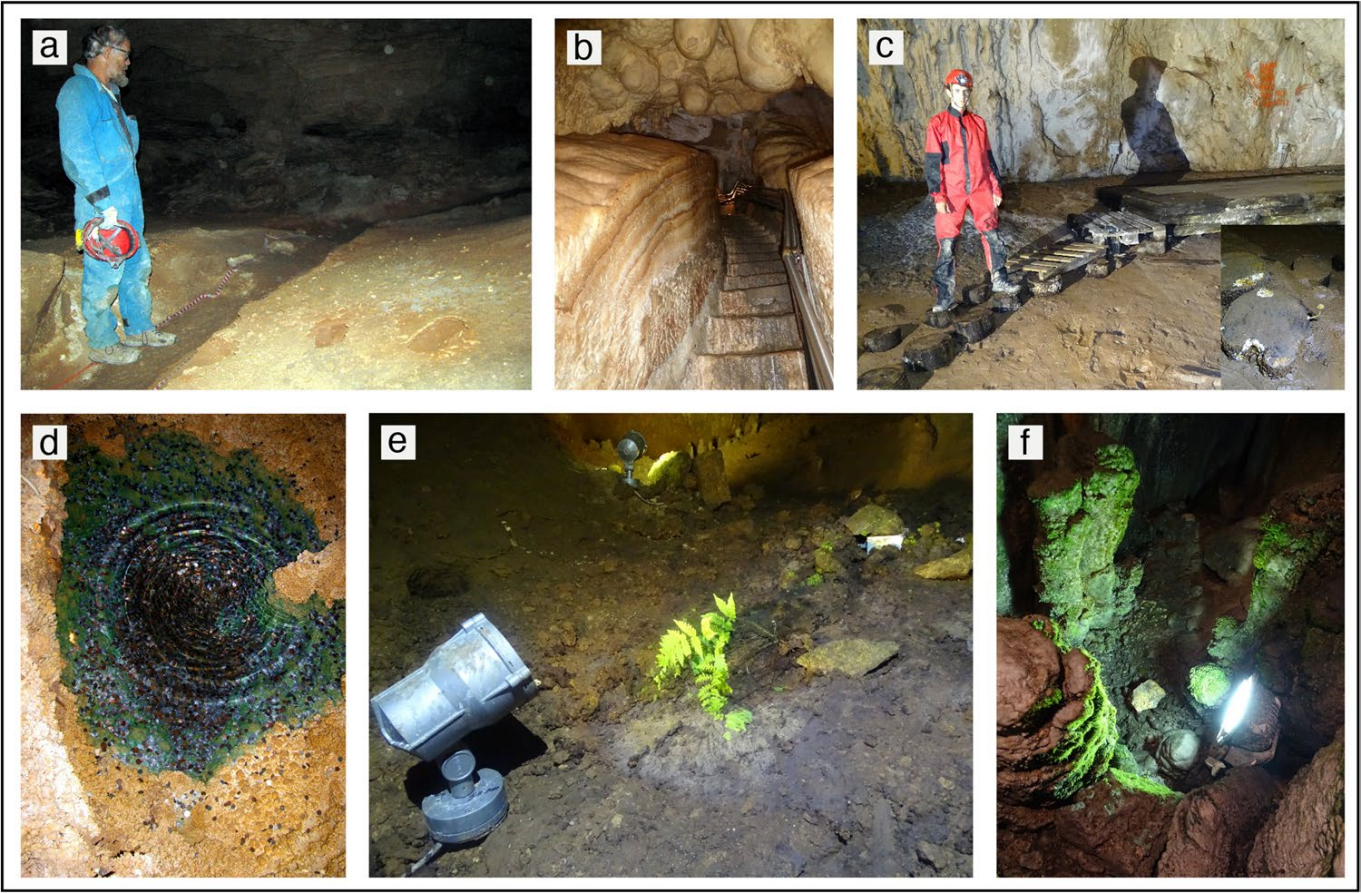
(**a**) A wild cave tour trail in Carlsbad Caverns, New Mexico (USA). The trail is clearly visible, but note the two footsteps in loose sediments to its right (photograph by Jo De Waele); (**b**) steps cut into a thick flowstone in Toirano Cave, northern Italy. These “heavy” works were carried out in the 60 s (photograph by Philippe Audra); (**c**) Wooden walkways in Bijambare Cave, Serbia. The small inset on the right shows fungi on the wooden trunks (photograph by Jo De Waele); (**d**) a pool with hundreds of coins, which have created a toxic environment, in Sonora Caverns, Texas (USA). The green colour derives from copper oxides (photograph by Jo De Waele); (**e**) Fern (Pastena Cave, central Italy); and (**f**) bryophytes (Collepardo Cave, central Italy) growing on speleothems close to lights (photographs by Jo De Waele)

Besides surface activities which can indirectly modify the cave environment (e.g. modification of the catchment basin, groundwater, and soil pollution, etc.), when caves are opened to speleologists and/or tourists an additional impact is produced (e.g. Calaforra et al. 2003). The mere passage of people causes the original surfaces to be touched, leaving foot- and handprints (Fig. 5a). In addition, in some show caves, to allow a comfortable progression cave sediments or speleothems have been sometimes cut down permanently, and natural cave passages have been enlarged modifying the original morphologies (Fig. 5b). Another impact produced by humans entering a cave is the modification of the cave atmosphere composition and microclimate. Indeed, the human body temperature releases energy into the cave, breathing causes an influx of CO_2_, and clothes and skin introduce alien particles (lint, epithelial cells, bacteria, spores, and seeds, etc.) (Jablonsky et al. 1993; Balestra and Bellopede 2022). In addition, when a cave is also equipped with a lighting system and other infrastructures, the energy balance increases (more energy is released into the cave environment). Infrastructures introduce foreign materials (plastic, steel, wood, glass) which alter the natural nutrient-poor cave environment (Fig. 5c-d), and the artificial light creates the favourable environment for photosynthetic plants to grow (the problem of lampenflora) (Mulec and Kosi 2009; Mulec 2014; Baquedano Estevez et al. 2019), which also alters the nutrient availability in the cave (Fig. 5e-f). The microbiological impact caused by visitors must be considered too (Saiz Jimenez 2012; Mulec 2014). Attention towards this topic was first raised when, starting in the 60s, after artificial lights were installed, the famous Lascaux Cave got impacted by the growth of photosynthetic algae and cyanobacteria, and later by fungi and a variety of microbial communities (Bastian and Alabouvette 2009). In more recent times, these environmental concerns have increased drastically with the insurgence of the “White Nose Syndrome” in 2006, which decimated the bat population in northern America in a few years’ time (Blehert et al. 2009), and the present pandemic situation (COVID-19), which required the adaptation of strategies to avoid the spreading of the coronavirus (Barton 2020).

To sum up, caves, both the wild (i.e. caves non-equipped for tourist visits) and the tourist ones, are fragile and precious environments and must be protected for several reasons, in particular:

1) they are part of a vulnerable aquifer system; underground karst water is an extremely important drinkwater resource, but it can be polluted very easily and has no natural depuration processes that can lead to mitigation of any possible pollution (Moldovan et al. 2020; Ruggieri et al. 2017);

2) caves, and the fissures around it (the underground ecosystem) are extremely rich in biodiversity, hosting important endemic species, often relicts of past climate conditions, and geological landscapes and ecosystems (Mammola et al. 2016; Culver and Pipan 2019). Alongside the precious invertebrate community, caves are also often home to vertebrates, such as the olm (*Proteus anguinus*), the Texas blind cave salamander (*Eurycea rathbuni*), or bats, just to mention the most important ones. Bats are protected by international laws, and their utility in the natural environment (most species being insectivorous) is beyond any doubt. In particular, hibernation colonies and breeding roosts of these mammals are extremely vulnerable to human presence. If present, the caves should be protected and closed to any visits (Medellin et al. 2017);

3) caves are also important geological and archaeological repositories, in which fossil bones and different archives of past climates and environments are hosted and preserved. Most of these deposits are still to be studied and must be protected from destruction for future studies. Caves were often used by animals, including our human ancestors, as shelters, burial places, or living quarters. These deposits are often protected from the external processes (wind, erosion) by the cave environment, and activities inside the caves can alter their original state of deposition (Gillieson 2011).

## Evolution of show cave management

In the early days of cave tourism, adaptations to the underground environment were limited, and visiting a cave was often an adventure relying on local guides involving complex logistics with many people assisting the, often, “honourable” and “noble” guests. Travellers in those years were generally of important and wealthy families (including kings and governors) or writers, with sufficient financial possibilities to cope with travel expenses and all kinds of costly side activities. Travelling to the caves and their karst areas often involved strenuous trips across mountains with poor connections, involving a certain number of supporting staff, and this type of leisure was restricted to few (wealthy) people. The visits of important people to caves, however, pushed local stakeholders and governors to invest in the caves and their surroundings, thus leading to the construction of more accessible roads, the first ground facilities close to the entrances, and internal pathways and stairs, or even electric lights. But prior to WWII, most caves were scarcely adapted, and one of the attractive things of cave tourism was the “sense of adventure”, with most caves accessible via more or less well laid-out paths both to get to the entrance, and into the cave. Many of these early cave exploitations were family based, with no real urge to be economically rentable: the income of visitors to the cave was often a welcome “extra” in the family budget (Gauchon 1996). If we count these caves as “true show caves”, in several European countries there were more show caves than there are today.

The industrial revolution increased the transport infrastructure, and thus the mobility of more people, in turn increasing the number of visitors to the caves. Several show caves became real commercial enterprises, in which the number of visitors had to increase to drive up the economic benefit, and this required larger paths, easy access and walk-through-the-caves, and new infrastructure both outside and inside the cave. Many of the family-driven caves were progressively abandoned, being unable to compete with the fewer but larger show caves. Abandonment of earlier show caves was related to three main reasons (Gauchon 1996): 1) remoteness and difficulty in access: as visitors were getting used to easy access roads and cars (or trains), caves reachable only after long walking through the mountains were progressively skipped in favour of caves close to communication ways. 2) Progressive loss of naturalistic appeal: the early visits often were without guides, and visitors often took “souvenirs” from the caves, resulting in a loss of aesthetic value and in a progressive visitor number decrease. 3) Many of the earlier show caves were connected to the thermal baths, and with the decline of the thermal business, also many show caves nearby lost their main source of visitors. 4) Some caves, after a period of activity, were declared economically not-sustainable. Income from visitors did not cover the management costs.

Unfortunately, some of these abandoned show caves have undergone great and irreversible damages (especially if they were easily accessible, with paths, and left uncontrolled) and are truly lost for future uses. Other caves have been equipped but were never opened to public for a variety of reasons: in general, these infrastructures required an investment to be removed, so pathways, electric light systems, etc., were left in place, doomed to decay over short time periods (Gauchon 1996).

Despite these failures, during the last 50 years or more, show caves have become a reliable source of investment and income, a true “cave business” (Cigna and Forti 2013), with a rapidly rising trend until the 80s. In more economically developed countries (e.g. Europe, North America, Japan, South Korea, and Australia-New Zealand), after the boom in the late 70s–early 80s, numbers started decreasing, pushing show cave managers to raise the quality of their tourist offers (Biot and Gauchon 2005). Visitors are increasingly becoming more culturally demanding, and they have a growing desire to increase their knowledge on karst, the natural and wonderful world of caves, their biodiversity and geodiversity. This has led to the creation of networks of show caves, and the participation of show cave guides in refresher courses on the multiple aspects of cave science. The opening of new show caves, the increasing quality in their management, and better transport infrastructures, started to invert this negative visitors’ trend in the beginning of the third millennium for some of the better managed caves. An increasing number of caves are being opened to public in developing and least developed countries, such as Mexico, Brazil, Thailand, and China. The economic and cultural gap in these countries is leading them to make the same mistakes made by early cave managers in the USA and Europe.

Caves can have different types of ownership and management: they can be public (municipalities, states, federal agencies) or privately owned. In both cases, they can be managed by public authorities (especially the caves falling under the management of Nature Protection administrations such as Parks), by private organisations (even at a family level) or have a contracted management. In all cases, there is always a conflict between conservation and tourism exploitation, and generally the economical return is being considered a priority as compared to natural heritage conservation and management. Only the biggest show caves, and those managed (and financed) by public stakeholders, can afford to invest in high-quality infrastructures, modern lighting systems and monitoring networks. Some of these also devolve part of their income to scientific research and development, and to the training of cave guides. Smaller show caves prefer (or are obliged) to invest only in tourism development, advertising, and safety of both guides and visitors. In economically developed countries, however, the greater networking (allowing guides to have easier ways of getting trained) and importance of public opinion (with visitors evaluating the visited sites, and social networks increasing the visibility of such polls), and the greater density of show caves, pushes cave managers to raise the quality of their offer. Earlier bad experiences of the recent past (show caves that have been closed because of irremediable damages, or other caves that were opened for a few years but soon abandoned because economically unsustainable) aid in making managers understand that they have essentially non-renewable resources at hand. Unfortunately, the same conditions do not occur in developing and least developed countries. In these areas of the world show cave development and management is often poorly controlled, and this “wild” development often resembles to what happened in the 70s in Europe and the USA, generally with detrimental effects on the cave and karst environment.

## Existing guidelines for the protection of cave and karst environments

Cave and karst protection policies are generally defined on a national, regional or local scale, and differ from continent to continent, between countries and even within single nations. The most comprehensive guidelines on cave and karst protection are reported by the International Union for Conservation of Nature (IUCN) (Watson et al. 1997; Gillieson et al. 2022). These indications are often used by legislators worldwide as the starting point for defining the protection framework and the local management practices in these delicate areas. Caves can be protected by the creation of natural reserves (i.e. National and Regional Parks), or directly by creating a cave-dedicated legislation that considers all actions threatening cave conservation (e.g. speleothem collection, illegal waste disposal, etc.) as a criminal offence (Middleton 2016). Cave environments can also be “indirectly” protected through the inclusion in the UNESCO World Heritage List: indirectly because this list does not assume further environmental protection, since all the proposed UNESCO sites must already be protected areas, but it raises awareness towards the importance of this unique environment. In addition, an international tool to protect endangered fauna and flora is represented by the institution of the IUCN red list of endangered species, which is recognised to be the most comprehensive, objective global approach for evaluating the conservation status of flora and fauna (Rodrigues et al. 2006).

In the USA, the Federal Cave Resources Protection Act, enacted in 1988 (Huppert 1995), had the purpose of protecting “significant caves on Federal lands”, with ambiguity regarding the true meaning of “significant”, and the fact that only caves in Federal land might get protection. Fortunately, all caves falling in National Parks are considered “significant” and thus fall under the protection of this law. A major drawback of this law, however, is that only the cave is protected, regardless of its surroundings (the karst landscape around and the catchment basin). Many show caves are on private lands and have no legal protection at all. In the same years, another large country with important karst areas (Brazil) started to enforce laws regarding cave and karst protection, especially related to the growing mining industry. Directive 887 (1988) of the Brazilian Institute of Environment and Renewable Natural Resources (IBAMA) included caves, as well as lakes, rivers, and other natural resources, as “State properties”, and therefore, they should be included in environmental studies and protected (Auler and Piló 2015). This law brought to the creation of the inventory of caves all over the country, and to further laws to define the significance of caves, since not all caves could be integrally protected. In fact, cave protection in this country severely interferes with the ore and mining industry, one of the greatest economic sectors in this country. Unfortunately, in 2022 the Brazilian Government has changed this law seriously putting in danger the many caves hosted in host rocks of economic interest, which will now be viable to commercial exploitation (i.e. mining and quarrying) (de Oliveira et al. 2022).

The most important international directive regarding the protection of cave environments is the Council Directive “Habitat” n. 92/43/CEE of the European Union. It provides guidelines to protect and/or restore different habitats and species (animals and plants) of Comunitary Interest identified in the same document, through the creation of a network of protected areas (Natura 2000; art. 3) and the promotion of scientific research (European Commission 1992). Habitats are grouped in Annex 1, which includes “caves not open to the public” in the group “Rocky habitat and Caves” and corresponding to the code 8310. In addition, annex II of Directive 92/43/CEE identifies “Animals and plant species of Comunitary interest whose conservation requires the designation of special areas of conservation”, a list that includes several species specialised to cave environments (e.g. bats, several amphibians, etc.). This Directive, although essential in protecting caves with important zoological and botanical assets (since it essentially protects animal and botanical species and their habitats), does not directly protect caves in which these biological values are not strongly represented, such as caves with unique morphologies, speleothems, or sedimentary archives.

**Fig. 6:**
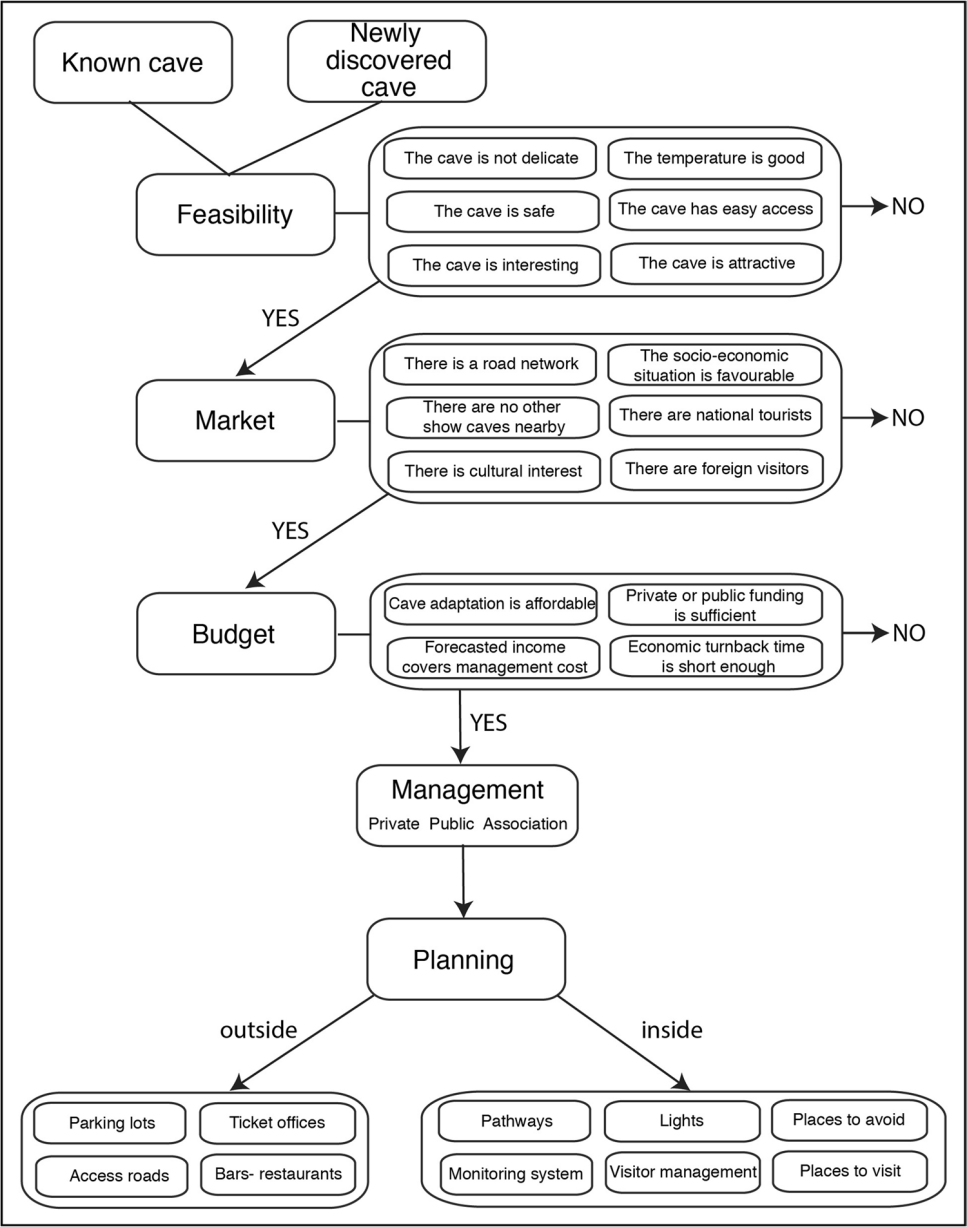
Flowchart showing the various steps that are required to develop a natural cave into a well-managed show cave. See text for details

At present, the only important official document produced by international associations regarding guidelines for cave frequentation is related to show caves and was issued by the International Show Cave Association (I.S.C.A.) and the International Union of Speleology (U.I.S.) in 2014 (ISCA 2014). This document does not represent legal requirements but aims at providing indications as “best practices” regarding the creation of new show caves and the management of caves open to public. These international guidelines are based on the expertise of the caving community and the experience of show cave management all over the world. Despite the existence of these guidelines, even in the recent past, not all show caves have adopted these good practices, and poor attention has been paid to cave environments during the opening of caves to tourists, because of a lack of awareness towards the fragility and the scientific, geological, and ecological importance of these environments. As a result of this, a strong impact was often produced in these poorly managed caves. However, in recent years, owners and managers of show caves have been realising that it is of primary importance to keep the show cave as clean, natural, and beautiful as possible, in order to ascertain its touristic appeal also in the future and for many generations to come. A show cave can be an important source of income also creating a wide range of secondary economic activities and should therefore be managed in a sustainable and endurable manner. A summary of these guidelines is reported below and is schematically reported in Fig. 6.

### Choice of the cave to be opened to the public

Not all caves can be eligible as show caves or places suitable for speleological guided trips. The suitability of a cave to be subjected to mass tourism or occasional speleological guided visits depends on a series of factors, including its location, its physical, biological, and environmental characteristics, and the economic viability of a potential tourist adaptation and related use as a show cave.

A true show cave must be adapted for the frequentation by groups of visitors. Such caves are generally large and more or less horizontal, so that there is no need to overcome large vertical drops or pass through long narrow passages. To be attractive for tourists these caves must also be beautiful, with rich and pristine speleothems, or special natural shapes and characteristics (e.g. river, waterfalls, nice cave wall morphologies, etc.) that may be of interest to the visitor. These characteristics can also include archaeological, palaeontological, and cultural heritage of high value (at least at a regional scale). The physical adaptation of the natural pathway must not require too extensive enlargements, heavy infrastructure and costly operations, and must always be weighed against the potential economical revenue of the visits. Permanent damages (opening of passages, excavation of artificial tunnels, pumping of flooded passages, etc.) must always be accompanied by an environmental impact assessment.

Certain caves (or cave passages) are not suitable to be opened to tourists, like the ones hosting large roosting (breeding)/hibernating bat colonies, or cave chambers decorated with fragile speleothems which may be damaged by the installation of artificial walkways, or caves that are seasonally flooded, or vertical shafts with no appreciable horizontal passages, just to name a few examples.

Some caves that are not deemed suitable to be equipped for common tourist visits, as well as some non-equipped parts of show caves, can sometimes be used for speleological guided tours. These visits are carried out with suitable equipment (helmets, boots, caving suits) and can eventually require the use of special tools (vertical rope equipment), depending on the type of visit (normal cave or extreme visits). However, also these speleological visits require the installation of an obligate route (signed pathway) to avoid leaving foot- and handprints everywhere, with the aim of keeping the cave environment as pristine as possible. Nevertheless, speleological visits which do not require specific caving techniques (e.g. abseiling shaft) should always be preferred for safety reasons also, since cave rescuing operations are always complex. In all these cases (normal show cave tour or speleological visits) the presence of cave guides is mandatory, in order to have a control on the participants (both for their own safety and for the safeguarding of the cave environment) and give explanations during the visits.

### Scientific and speleological investigations

Once a specific cave has been chosen as a potential show cave and/or for guided speleological tours, it needs to be investigated in detail. This means that all these caves have to be explored (a cave is never fully explored, but all main branches of the cave need to be scouted with modern speleological techniques at least up to some technical obstacle (sump, rising shaft, boulder choke, etc.)). A detailed survey of the known cave needs to be produced (if not existing). These maps require sufficient detail to allow the planning and construction of the cave paths (both tourist and speleological) and of the electric lighting systems (if required), and the decision on where cave monitoring instruments need to be placed (see next paragraph).

Sufficient information about the scientific aspects of the cave needs to be available, including knowledge on archaeological deposits, palaeontology, geology, hydrology, and biology. This last branch is particularly important to understand whether there are reasons for limiting visits to the cave (or certain parts of it) because of the presence of endangered and vulnerable species (e.g. *Proteus anguinus* or olm) or bat colonies (reproductive summer or winter hibernation colonies). Prior investigations are also useful to determine which places should be part of the guided tours (beautiful corners, impressive speleothems, nice morphologies, scenic spots) and those places that need to be excluded from the future cave tour (places with delicate speleothems, chambers with large bat colonies or presence of delicate ecosystems, areas subjected to seasonal floods, etc.). If no such information is present, it is mandatory to perform such investigations prior to any action in the cave itself.

### Monitoring before opening and during show cave management

Caves are natural environments with relatively more stable conditions with respect to the external atmosphere. They can be classified in high-, medium-, and low-energy caves, depending on the amount of energy flowing through the underground passages (Gillieson 2011). The energy flow in a cave, in natural conditions, mainly depends on the flow of fluids (water and air) through them (Badino 2010), and these depend on the isolation of the cave from the external environment. Caves in which large water flows are introduced (sinking rivers, or caves with significant hydrological changes) are always high-energy caves. On the other extreme we find deep isolated karst voids with no natural entrance. The energy balance of a cave is also greatly influenced by its depth from the surface and the consequent presence (or not) of multiple entrances. Caves with multiple entrances located at different altitudes will be crossed by strong air currents, thus greatly influencing the underground micrometeorology. Local micrometeorology is also influenced by processes such as evaporation and condensation, since the thermal capacity of water is great and latent heat is significant (thus absorbing or releasing great energy fluxes where these processes are active).

Opening a cave for tourist or speleological visits introduces an artificial amount of energy, both directly and indirectly. Visitors introduce both heat, water vapour, and carbon dioxide, which must be considered when defining the possible carrying capacity of the cave (the number of visitors per time unit that is sustainable for the cave environment, i.e. allowing environmental parameters such as temperature and CO_2_ concentration in air to return to the initial natural values from one day to the other). Also, modifications to the cave morphology (opening or enlargement of entrances or cave passages) create changes in air flow, and thus energy flow, which can have great negative impacts on the underground environment.

Therefore, it is necessary to measure the cave air characteristics, and especially temperature (T), relative humidity (RH), and CO_2_ levels (De Freitas 2010). If these parameters are not monitored, then a possible long-term increase in cave temperature and cave air CO_2_ may occur, resulting in a potential impact on chemical and physical parameters (temperature, pH, etc.) of isolated water ponds as well, where even very small changes can interfere with the sustenance of any kind of lifeform. Not only the organisms living in water can be threatened by temperature and CO_2_ modifications, but also all the troglobiont species of a specific cave. Furthermore, in karst caves, temperature, as well as air flow modifications, can produce changes in moisture condensation and water evaporation, triggering the development of condensation-related morphologies, thus modifying the cave’s original aspect. Nonetheless, considering the essential role of carbon dioxide both in speleogenesis (in carbonate rocks) and in speleothem deposition, the long-term modification of its concentration can trigger carbonate speleothem corrosion (Baker and Genty 1998). It is thus clear that artificial modifications of cave microclimate parameters can have important negative consequences not only on cave ecosystems, but also on their intrinsic beauty, considerably lowering the appeal and the attractiveness towards visitors and causing both biodiversity, geodiversity, and economic loss.

Besides these parameters, also radon concentrations in air should be measured in each season of the year, at least for one year and in different areas of the cave, to see whether natural radioactivity related to this gas needs to be taken into account for the safety of the people working underground (cave guides). High levels of radon might cause exposure levels to radioactivity above those recommended for underground labourers (based on international and national regulations) (Gillmore et al. 2000; Cigna 2005). Finally, cave water should be analysed on a regular basis for the early detection of leakage of wastewaters from the tourist infrastructures above the cave.

General monitoring should be continuous, with measures every 30 min or an hour for T, RH, and CO_2_ and should last at least 12 continuous months (in order to reflect all seasons). Experiments should be carried out with groups of visitors of various dimensions, especially in places where cave guides give their explanations and visitors stay for longer times, monitoring the changes of all parameters and their return to natural values. These experiments will allow to define the carrying capacity of the cave, which should be taken into account during the management of the visits.

After the year of preliminary monitoring, during the normal management of the cave, measurements should be continued for T, RH, and CO_2_, with timing and placing to be defined case-by-case and based on the results of the first monitoring campaign.

### Management of cave visits

The number of visitors that can be allowed into the cave depends on a series of factors, mainly related to the dimension of the cave, the length of the underground visitor trail, and the environmental conditions of the cave itself. The safety of visitors is the main factor that needs to be accounted for, but visitor numbers should also take into account the carrying capacity of the cave, which has to be defined according to cave monitoring.

Each group of visitors should be accompanied by two cave guides, at least when relatively large groups are allowed into a cave: one of these will be responsible for the explanations and will guide the tour. This guide normally will be in front of the visitors and will make different stops in places suitable to give logistical, historical, and scientific explanations. The second guide will close the visitor group, making sure no one is left behind and controlling the correct behaviour of visitors. If tourists are not supervised by a cave guide, it is more likely that negative behaviours threatening the cave habitat occur, such as speleothem touching, people leaving the trails, introducing food and beverages, smoking, rubbish disposal and coin launching, leading to a visible degradation of the cave environment and introduction of alien microorganisms, causing darkening of speleothem surfaces and accumulation of rubbish and/or coins in natural spots.

Special consideration must be reserved for tourist caving tours. Indeed, several show caves around the world offer this type of visit in some wild portions of the cave, while some wild caves can be visited using speleological equipment and accompanied by a speleological guide. Unfortunately, internationally recognised guidelines do not exist for this form of tourism, but only some indications provided by national speleological associations (e.g. The National Speleological Society), local public authorities, or national governments. This type of tourism can have a strong impact on cave ecosystems since it is rarely regulated, and it is not limited to the artificial walkways but to the entire underground environment, exposing all cave surfaces to a direct interaction with visitors. Cave managers and/or public authorities should produce specific regulations for each cave including minimal impact caving codes (i.e. group composition, identification and delimitation of tracks, cave equipment maintenance protocols, forbidden activities such as smoking, etc.) in order to minimize the impact of speleological tourism.

### Logistics outside and around the cave entrance

Caves are an integral part of a landscape and, for those formed by dissolution, of a karst environment. Protection measures for show caves should not be limited to the cave itself, but must extend to the surface above the cave, the areas adjacent to the entrance, and ultimately, to the entire recharge area of the cave. Infiltration of waters must be assured: this means that construction above the cave footprint needs to be avoided, or at least surface runoff must be allowed to go underground through natural pathways, preventing flow to be too concentrated. Often the construction of large parking lots and buildings causes the creation of concentrated runoff, with negative impacts on the underground environment. It is often advisable to create parking lots and build infrastructures at some distance from the cave footprint and from the cave entrance, limiting the direct impact on the karst environment. In that case, a shuttle service may be a good solution, using electrically powered trains or buses, or creating a dedicated walking trail. All activities that create white and grey water need to be managed carefully, with a wastewater management that is sustainable in a karst aquifer area. Many techniques exist for treating waste waters (including natural systems), but decision needs to be based on the quantities of wastewater that is produced and logistical requirements.

### Materials to be used for in-cave infrastructure

Trails with the minimum impact on speleothems and water bodies are the most suitable to realise artificial paths in a show cave. The best material to be used to build artificial pathways is high-quality stainless steel: indeed, this material does not deteriorate over time and does not cause contamination in cave environments, and, if carefully planned, these infrastructures can be entirely removed from the cave (in case pathways are abandoned for some reason). Its counter indications are the rather high cost (for high-grade stainless steel), its relatively important visual impact, and the rather complicated installation (i.e. weight, assemblage, and welding). Although new plastics are becoming increasingly resistant and are much lighter and cheaper, their use should not be preferred to stainless steel since it introduces a potential source of contamination. Indeed, microplastic pollution is becoming more and more diffuse in underground environments, including karst groundwater systems (e.g. Panno et al. 2019; Balestra and Bellopede 2022). The cave walkway should be carefully designed and subdivided in portions which should be prepared before bringing them into the cave. The only process that should be carried out inside the cave is represented by the assemblage of the walkway portions in order to minimize the disturbance to the habitat, including bats, and pollution. For caves developing in limestone bedrock, concrete pathways may be considered. However, since concrete is extremely difficult and expensive to be removed, it should be used with care over small portions of pathways or along relatively narrow tracks. Any type of organic material must be avoided. Even if wood is considered an environmental-friendly material to be used in natural areas, it does not belong to the cave environment. Indeed, wood in caves does not last long because of the high humidity, and it introduces organic material and microorganisms (bacterial colonies, fungi, moulds) which are not naturally found in caves, thus altering the local microbial and invertebrate communities. Pathways in show caves must always be equipped with stainless steel handrails. This is needed to ensure visitor’s safety and to avoid them to exit the designed path. In wild caves opened to speleological tours and/or in wild portions of show caves where tourists can access with guides, paths should be identified as well and indicated with resistant reflective tape to avoid tourists to walk everywhere. Today also wires supported by small rods are used, having a smaller visual impact on the cave (Ayuso and Calaforra 2016). Long-lasting materials need to be used to avoid the introduction of microplastics and other foreign microparticles.

Caves are naturally dark environments, and any artificial light introduces strong modifications to their natural conditions. There is no need to light up the cave entirely: only important scenic spots might be highlighted for short periods, leaving most of the cave in its natural dark state. Permanent, low-level led lights are required only on the cave path, pointing to the floor and functioning as safety lights during the entire opening period of the day. The main cave lighting systems must release the lowest amount of heat as possible (i.e. light-emitting diodes—LED lights or cold cathode lamps—CCL), and light intensity should be kept as low as possible. Every light point must be set at least 2 m away from any surface, especially if wet and muddy (speleothems, cave walls, and floors) to inhibit the development of lampenflora. Permanently turned-on lights should be limited, preferring the realization of a system that can be switched on and off easily (with two independent circuits, one for the main lighting system, and one for the smaller and dimmed safety lights), in order to reduce the time of artificial illumination inside the cave. Note that electronics can often have trouble working well and over long times in the wet and harsh conditions of caves. The artificial illumination system should be monitored as well in order to identify the early development of lampenflora and take actions at the first evidence of its development (e.g. modifying the orientation of lights, their positioning, the light intensity, etc.).

### Cave guides

We define “show cave guide” the person leading a group of people along the paths of an illuminated show cave, and “speleo-guide” the person leading a group of people along signed but less equipped paths through a wild cave area (where no fixed lights are placed).

A show cave guide is a professional figure able to safely guide people along the cave paths and simultaneously provide correct information about cave ecology, geological features (e.g. speleothems), cave formation (i.e. speleogenesis), cave conservation, historical, and/or archaeological features of a cave. It should be someone with good communication skills, able to involve tourists and introduce them to this underground world providing correct information about cave habitat and its fragilities. A show cave guide should also have basic knowledge on safety measures while leading people along artificial paths in relatively safe environments (presence of paths, lights, etc.).

A “speleo-guide” should have the same cultural background and communication skills of a show cave guide. However, this figure is far more specific, guiding tourists outside the artificial paths of a show cave, leading people to explore hidden treasures which can sometimes not be easy to reach. Thus a “speleo-guide” should be an expert caver combining good knowledge of cave habitat and technical skills. Besides providing correct information to tourists, a profound knowledge of the cave environment is a must for a speleo-guide, since leading tourists outside the artificial paths using proper equipment (cave helmet, suit, boots, etc.) can have a strong impact on both cave habitat, its conservation, and tourist safety.

## Materials and methods

Our investigation is primarily based on the private website www.showcaves.com. The authors have carried out an Internet search for all these caves, often finding dedicated websites where information on the caves, entrance fees, and curiosities is readily available for external use. Where no website was available, different travelling information websites (e.g. tripadvisor, trip, Tong Cheng, Xin Xin Travel, etc.) were used to get an idea of entrance fees, popularity of visited show caves, and geographical information. Simple independent Internet searches using key words such as “show cave”, “commercial cave”, were also carried out to find the caves listed in Dukeck’s lists, but also to discover caves that were not reported in his lists. These Internet investigations have taken place between May and June 2021 and enabled 95% of the listed caves to be traced and consulted. The list of show caves was then divided into countries, and these shortlists were sent to prominent cave and karst researchers or cavers of 30 countries (the ones with more than 10 show caves listed) to check completeness and reliability of data. Accuracy of the entrance fees (based on values relative to 2019–2021 and their exchange rates in June 2021), cave name, and location is judged very high, since this information is reported in good detail for over 95% of all caves. Regarding entrance fees, whereas in most show caves (95%) the visits are of one type (thus one entrance fee), we normally took the fees of a single adult (and not that of children, family discounts, or groups), thus overestimating the income deriving from ticket sales. In about 5% of the caves multiple visits are offered, from long and shorter tours, to combined tickets, or adventure (“wild cave”) tours. For our research we took the normal, most chosen, guided tour as an indication for the total income from tickets.

Also, the length of the show cave path is less precise but corresponds to the true internal cave paths for 70% of all listed caves. In several caves the cave path length could be estimated based on the time of the visit (which is often reported as a practical information for visitors). The less precise information is related to the total length of the caves (50% is based on registered and certified data, in countries where cave registers exist and are freely available) and, especially, number of visitors per year, a figure that is often not reported (only 10% of all show caves has a reliable estimate of yearly visitors for pre-COVID-19 conditions, whereas another 10% has been estimated by local collaborators and can be judged as being rather reliable). For some countries, and the most visited caves, these visitor numbers are relatively reliable but become less precise for smaller and less visited caves. The yearly visitor number, in these cases, is estimated based on a combination of web-based information (tripadvisor comments, picture posts, etc.) and cave proximity to populated areas or transport systems (railroads, highways, tourist routes). Unfortunately, there is no other efficient manner of getting correct numbers in most cases, but for our global analysis they give a good (although rough) idea of global visitor numbers and, consequently, incomes from entrance fees.

In this paper we regarded show caves having a combination of at least two out of three criteria: 1) some kind of adaptation of the entrance and/or of the internal path, to make the cave easily accessible to most people (entrance gate or door, artificial entrance, stairs or elevators, delimited pathways often with handrails, lighting system); 2) an “official” ticket has to be paid to enter the cave at some kind of ticket office (not to an improvised guide only); 3) visits are generally carried out accompanied by an official and recognised guide (who normally gives some explanations and takes care of visitor’s safety) or can sometimes be self-guided along fixed and clearly delimited underground paths, with or without an audio-guide or a leaflet (e.g. Carlsbad Caverns in the USA, Nerja Cave in Spain). Most of these caves are advertised in Internet, and by other means, thus they can be easily discovered by travellers. Especially in developing and poorly developed countries show caves can be rudimental, but as long as trails are clearly set out, a ticket (fee) needs to be paid, some kind of equipment is given to visitors (helmets, lights), and the visit is with a guide we considered these caves as “show caves”. Truly self-guided cave tours in poorly modified caves, not closed by gates, and without guides or equipment given to visitors have been excluded, even if a fee is paid to enter these caves.

For all caves geological information has been provided and the type of rock hosting each show cave has been indicated. To allow for analyses about the presence of show caves in relation to the hosting rock, the geological data have been subdivided into 7 major categories: 1) carbonate rocks (clastic and organogenic limestones); 2) evaporite rocks (halite and gypsum); 3) intrusive rocks (e.g. granites); 4) quartzite and quartz sandstone rocks; 5) volcanic rocks (ignimbrites and basalts); 6) true calcitic and dolomitic marbles (thus the high-grade metamorphic carbonate rocks, excluding meta-limestones and meta-dolostones); 7) artificial caves (i.e. reproductions of the original cave which is not visitable for conservation reasons). Show caves in conglomerate rocks, being rather rare, have not been considered here and were classified under the carbonate class (since these caves often are formed by conglomerate rocks composed of limestone or dolostone pebbles in a carbonate cement).

Considering that the main process leading to cave formation involves the action of water on soluble rocks, the extension of karst outcrops in each country has been investigated and put in relation with the number of show caves. For this purpose, a map representing the global distribution of karst areas was required. Two GIS (Global Information Systems) datasets exist and have been developed, respectively, within the KROW Project (Hollingsworth 2009) and the WOKAM Project (Chen et al. 2017; Goldscheider et al. 2020). The KROW Project (Karst Regions of the World) was dedicated to the creation of a database of karst distribution and biodiversity. It was developed by the Nature Conservancy and the University of Arkansas (USA) aiming at producing a worldwide map including global distribution of karst habitats and species. In this map, exposed carbonate karst (limestones and dolomites), evaporite karst (gypsum and halite), and pseudokarst are represented. The reliability of KROW is dependent on the accuracy of literature and reference data and the intrinsic error introduced during the digitalisation of maps at different resolution; in addition, data related to evaporite and non-carbonate karst outcrops in the Asian countries are to be considered less documented (Hollingsworth 2009). The WOKAM Project (World Karst Aquifer Map) was mainly focused on groundwater resources associated with karst reservoirs and the creation of a global map of karst aquifers developed by the International Association of Hydrogeologists (IAH) Karst Commission and supported by both IAH and UNESCO and is based on the Global Geological Map (GLiM) developed by Hartmann and Moosdorf (2012) (Chen et al. 2017). The world karst aquifer map, developed in the framework of the Worldwide Hydrogeological Mapping and Assessment Programme (WHYMAP), includes both carbonate and evaporite outcrops worldwide. Note that this map considers the aquifers, which means it takes into account also the carbonate and evaporite units that have no outcrops. However, as for the KROW dataset, its reliability is spatially variable, and inaccuracies may be present. In this paper, we compared the two datasets to identify eventual discrepancies. To calculate karst percentage for each country the TM_WORLD_BORDER-0.3 shapefile map was used (http://thematicmapping.org/downloads/world_borders.php) in association with WOKAM, whereas for KROW datasets, the shapefile world border map provided by the author was used. Both WOKAM and KROW shapefiles were used to identify countries where there are carbonate rocks, but which do not have show caves, thus aiming in finding places where, although caves could potentially be found, this form of tourism is not yet exploited.

All caves have been geographically positioned using available data in Google Earth and Google Maps, and when the location was not available, cave coordinates found on different websites have been reported in Google Earth. The location of each cave can thus be considered reliable, although a certain degree of uncertainty must be considered, depending on the accuracy of coordinate data found in Internet which cannot be verified. To create maps and spatial analyses, the open-source software QGIS3.16 was used. Population, country surface, and GDP(PPP) per capita (i.e. the gross domestic product at purchasing power parity) data were collected using the website www.worldometer.info, which is run by an international team of developers, researchers, and volunteers without any political, governmental, or corporate affiliation and which is largely used in several research fields. Caves have been grouped per country, and the total estimated yearly visitors of show caves have been compared with data about yearly international tourist presence downloaded from the United Nations Statistic Division of the Department of Economic and Social Affairs (https://unstats.un.org/unsd/snaama/downloads). When available, data related to the year 2019 have been used. In a few cases it was necessary to use data from 2018, 2017, and 2016 (about 22% of all countries with show caves) since data from 2019 were not available. Countries have been subdivided into 3 main categories to identify the wider potential tourism basin (if domestic or international): 1) countries with yearly international tourist presence greater than the country’s population; 2) countries with international tourism presence higher than 50% of the country’s population; 3) countries with international tourism presence lower than the 50% of country’s population. Data have been analysed on both country and regional basis. For this purpose, countries with at least one show cave have been further grouped into geographical regions, in particular: 1) Europe; 2) Africa; 3) Middle East; 4) Central and Eastern Asia; 5) South-Eastern Asia; 6) Oceania; 7) North America; 8) Central America; 9) South America. Pearson correlation coefficients have been calculated between the number of show caves and country GDP(PPP) and karst surface calculated using both WOKAM and KROW datasets. The results have been compared with data related to international tourism presence, ticket income, and country GDP(PPP).

## Results: analysis of show caves of the world

**Fig. 7:**
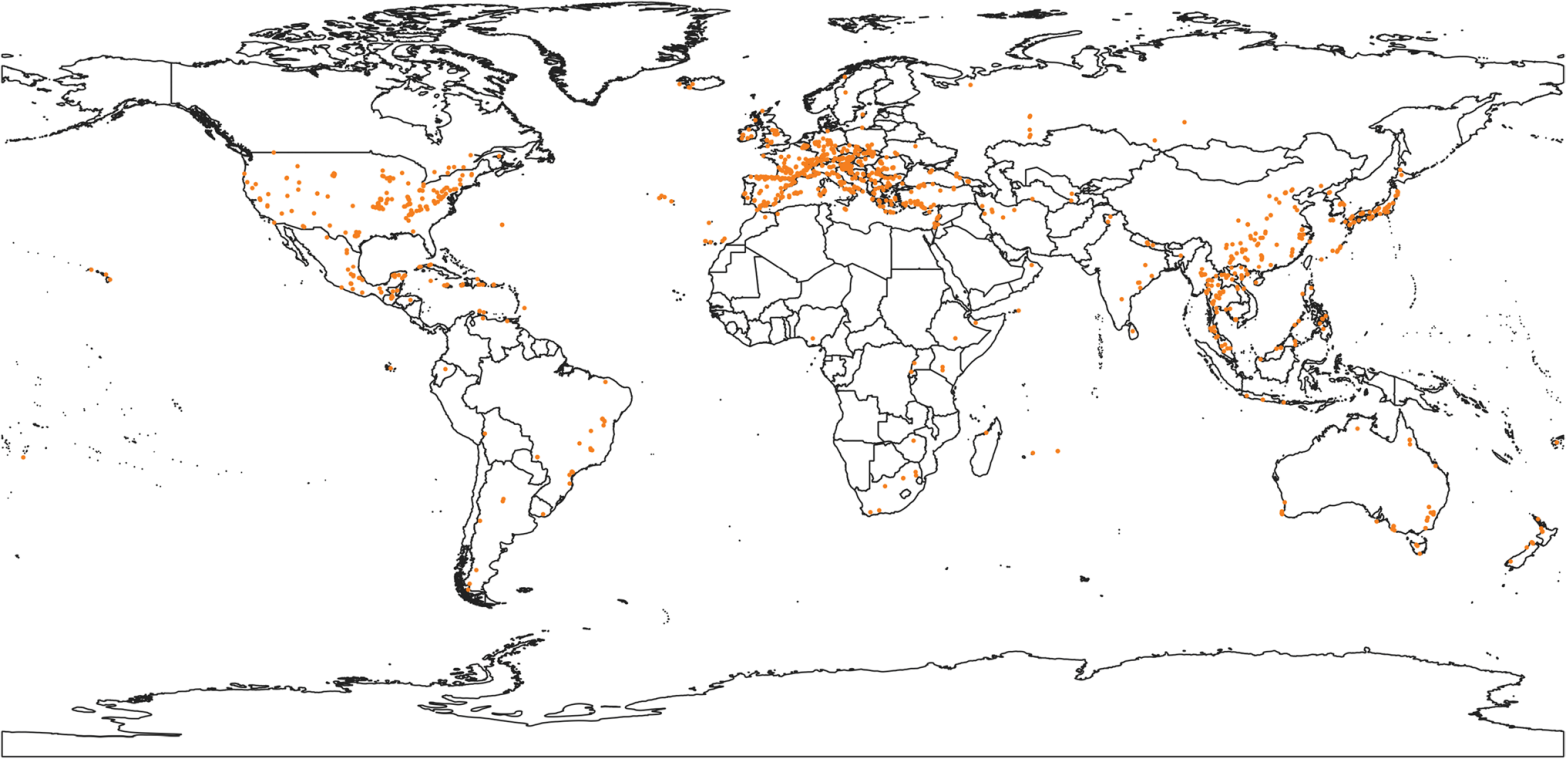
Global show cave distribution (orange dots)

A total of 1223 show caves have been identified in 95 countries over the world (Fig. 7; Tab. 1; and Tab. 2).

**Table 1:** Data from all the show caves identified within this work

Number of countries with show caves	Total number of show caves	Total show caves pathways (km)	Total cave length (km)	Total visitors in 2019 (million people)	Total income from entrance fees (million Euros)
95	1223	712.3	5,765	79.3	806.23

**Table 2:** Countries with show caves in the world. Data about population, GDP (PPP)/capita, and country surface are from www.worldometer.info. Karst surface percentage is expressed with respect to each country surface and is calculated using both KROW and WOKAM datasets. Additional data about show caves in each country are given in Online Resources 1 and 2

Geographic Region	Country	Population (million)	GDP(PPP)/capita (Euros) (*)	Country surface (10^3^ km^2^)	KROW karst Surface (%)	WOKAM karst Surface (%)	N° of Show Caves	Total visitors per year 10^3^	Total show cave paths (m)	Ticket income 10^3^ Euros
Europe	Austria	9.0	45	82.4	21.78	28.14	32	596.2	18,280	7707
	Belgium	11.6	41	30.3	8.89	31.16	14	796	8330	7947.5
	Bosnia and Herzegovina	3.3	11	51	40.35	77.32	4	55	3220	185.5
	Bulgaria	6.9	17	108.6	46.68	27.16	12	300	8290	935
	Croatia	4.1	22	56	46.34	49.34	19	330	4320	2141
	Cyprus	1.2	30	9.2	0	0	1	5	250	15
	Czech Republic	10.7	31	77.2	3.55	18.97	15	940	9030	6240
	France	65.2	36	547.6	34.28	49.63	105	5206.5	50,990	62,994.9
	Germany	83.8	43	348.6	5.53	21.17	46	2479.5	21,285	16,551.5
	Greece	10.4	24	128.9	43.58	40.15	26	555	8425	3777.5
	Hungary	9.7	24	90.5	15.62	9.91	13	552.4	10,070	3654
	Iceland	0.3	46	100.3	0.35	0	4	27	2600	2300
	Ireland	4.9	63	68.9	42.90	46.47	6	440	3480	5155
	Italy	60.5	34	294.1	43.27	29.29	53	2361	28,595	30,541
	Malta	0.4	34	0.3	100	74.66	3	25	280	975
	Monaco	0.04	60	0.001	100	18.76	1	50	100	250
	Montenegro	0.6	19	13.5	59.55	85.24	1	50	600	545
	North Macedonia	2.1	13	25.2	30.01	20.36	1	20	425	160
	Norway	5.4	51	365.3	2.29	2.99	1	10	400	130
	Poland	37.8	25	306.2	16.93	14.91	11	810	3450	2830
	Portugal	10.2	27	91.6	8.94	4.20	12	354.5	4165	2451.2
	Romania	19.2	22	230.2	10.37	11.42	21	541.5	10,760	1268.5
	Serbia	8.7	13	87.5	14.94	20.10	10	225	6860	581.5
	Slovak Republic	5.5	27	48.1	27.26	21.13	18	840	10,949	7990
	Slovenia	2.1	30	20.1	54.15	79.24	22	1128	13,862	26,138.5
	Spain	46.8	32	498.8	23.40	35.94	70	2932.1	32,980	34,177
	Sweden	10.1	42	4103.4	2.28	3.56	2	90	300	1240
	Switzerland	8.7	55	39.5	6.21	35.16	9	360	5560	5385
	Ukraine	43.7	7	579.3	42.77	41.94	3	1300	2550	530
	UK	67.9	37	241.9	21.71	27.36	22	1880	12,290	29,947.5
Africa	Algeria	43.9	13	2381.7	9.56	15.82	1	20	600	20
	Ethiopia	115	1,6	1000	34.11	29.08	1	5	500	25
	Kenya	53.8	3	569.1	15.45	3.08	2	15	200	290
	Madagascar	27.7	1	581.8	16.42	13.64	1	2	1000	40
	Mauritius	1.3	18	2	10.71	0	3	70	1400	255
	Morocco	36.9	8	446.3	26.52	36.18	2	60	600	70
	Nigeria	206.1	5	910.8	15.89	30.80	1	10	350	65
	Rwanda	13.0	2	24.7	2.49	0.69	1	1	1000	40
	Somalia	15.9	1	627.3	12.74	44.57	1	5	100	100
	South Africa	59.3	11	1213	16.20	5.14	7	495	3765	3520
	Uganda	45.7	2	199.8	2.11	1.88	1	5	100	10
	Zimbabwe	14.9	2	386.9	6.44	3.77	1	10	800	80
Middle East	Iran	83	17	1628.6	17.43	55.04	6	630	5400	6490
	Israel	8.7	32	21.6	50.00	69.43	2	60	280	393
	Jordan	10.2	8	88.8	21.15	55.90	1	5	200	5
	Lebanon	6.8	12	10.2	94.66	95.25	6	370	3165	3424
	Oman	5.1	34	309.5	5.13	27.39	1	75	860	1125
	Turkey	84.3	23	769.6	29.18	21.24	30	1701	0972	2212.9
	Yemen	29.8	2	528	29.41	53.00	1	5	2500	10
Central Asia	China	1439.3	14	9388	22.04	26.55	74	19,690	86,150	232,863
	Georgia	4	9	69.5	88.94	12.33	7	922	3825	4996.5
	India	1380	6	2973.2	15.00	11.99	9	2110	3730	1775
	Japan	126.5	35	364.6	5.37	0	60	1857	25,970	11,078.7
	Nepal	29.1	2	143.4	4.94	3.34	5	345	1620	1190
	North Korea	25.8	3	120.4	8.17	11.54	2	200	1300	150
	Russia	145.9	21	16,376.9	10.14	14.888	17	836.1	10,470	4361
	South Korea	51.2	32	97.2	6.02	6.07	12	2547	8800	11,959.8
	Sri Lanka	21.4	11	627.1	7.11	5.19	1	500	50	3250
	Turkmenistan	6	15	469.9	37.44	5.35	2	150	500	600
	Uzbekistan	33.5	6	425	49.91	6.58	1	500	70	500
South-East Asia	Cambodia	16.7	3	176.5	2.45	0.97	3	80	700	65
	Indonesia	273.5	10	1811.6	17.97	15.98	4	490	850	2960
	Laos	7.3	6	230.8	25.50	9.04	8	460	4860	420
	Malaysia	32.4	24	328.6	3.43	11.07	16	295	10,940	1727.5
	Myanmar	54.4	5	653.3	31.23	12.87	12	955	3900	940
	Philippines	109.6	7	298.2	16.21	26.21	9	380	2100	2430
	Thailand	69.8	15	510.9	6.23	6.45	26	1510	14,400	2839
	Vietnam	97.3	6	310	10.05	27.61	14	1505	7900	12,665
Oceania	Australia	25.5	41	7682.3	6.37	5.06	48	1315	20,785	23,324
	Fiji	0.9	8	18.3	0	14.20	1	20	100	120
	New Zealand	4.8	34	263.3	3.01	23.82	12	532	7100	17,839
	Tonga	0.1	5	0.7	0	0	1	5	400	10
North America	Canada	37.7	38	9093.5	16.46	16.63	7	180	1235	2725
	Mexico	128.9	15	1944	10.79	25.41	29	912.3	28,165	11,987.6
	U.S.A	331.0	50	9147.4	16.59	23.65	149	9965	117,480	152,541
Central America	Barbados	0.3	15	0.4	19.14	80.46	1	150	1600	6000
	Belize	0.4	7	22.8	22.78	51.53	1	10	400	200
	Bermuda	0.06	35	0.05	0	0	3	205	1100	4000
	Cayman Islands	0.07	40	0.2	0	0	1	20	1000	680
	Cuba	11.3	12	106.4	75.98	45.71	5	280	3400	1500
	Dominican Republic	10.8	13	48.3	38.14	24.30	4	70	1090	250
	Guatemala	17.9	7	107.2	30.74	34.99	5	120	2400	427
	Haiti	11.4	2	27.6	57.77	9.60	3	25	1200	40
	Honduras	9.9	4	111.9	17.41	6.40	1	20	400	20
	Jamaica	3	7	10.8	69.86	63.06	3	25	2030	400
	Puerto Rico	2.9	35	8.9	21.55	16.65	2	60	400	830
South America	Argentina	45.2	17	2736.7	5.17	1.62	5	135	520	533
	Aruba	0.1	33	0.2	0	0	3	25	210	143
	Bolivia	11.7	6	1083	3.92	6.79	1	30	660	120
	Brazil	212.6	13	8358.1	4.03	3.66	23	309	9880	1960.7
	Chile	19.1	20	743.5	23.93	8.74	1	50	200	475
	Curaçao	0.2	-	0.4	0	0	1	20	100	140
	Ecuador	17.6	10	248.4	12.37	13.50	2	20	2900	50
	Uruguay	3.5	19	175	0	0	1	10	100	20
	Venezuela	28.4	2	882	6.04	6.53	2	110	2700	1100

(*) Gross Domestic Product (GDP) per capita at Purchasing Power Parity (considering the relative cost of living) relative to 2017

Around 712 km of cave pathways has been visited by about 79.3 million people in 2019 and producing an income of almost 800 million Euros in the same year (Tab. 1).

The majority of show caves is hosted in carbonate bedrocks (93.62%). They are followed by show caves hosted in volcanic rocks (3.27%) and evaporite rocks (1.39%), while only few caves are found in intrusive rocks, quartzites and quartz sandstones, and marbles; only 3 caves in the world have been artificially reproduced to safeguard the archaeological content (mainly rock paintings) of the original ones (Tab. 3).

**Table 3:** Show cave subdivision according to the hosting bedrock

Hosting bedrock	Total number of show caves	Total % of show caves
Carbonates	1145	93.62%
Evaporites	17	1.39%
Intrusive rocks	7	0.57%
Quartzites and quartz sandstones	6	0.49%
Volcanic rocks	40	3.27%
Marbles	5	0.41%
Artificial	3	0.25%

**Fig. 8:**
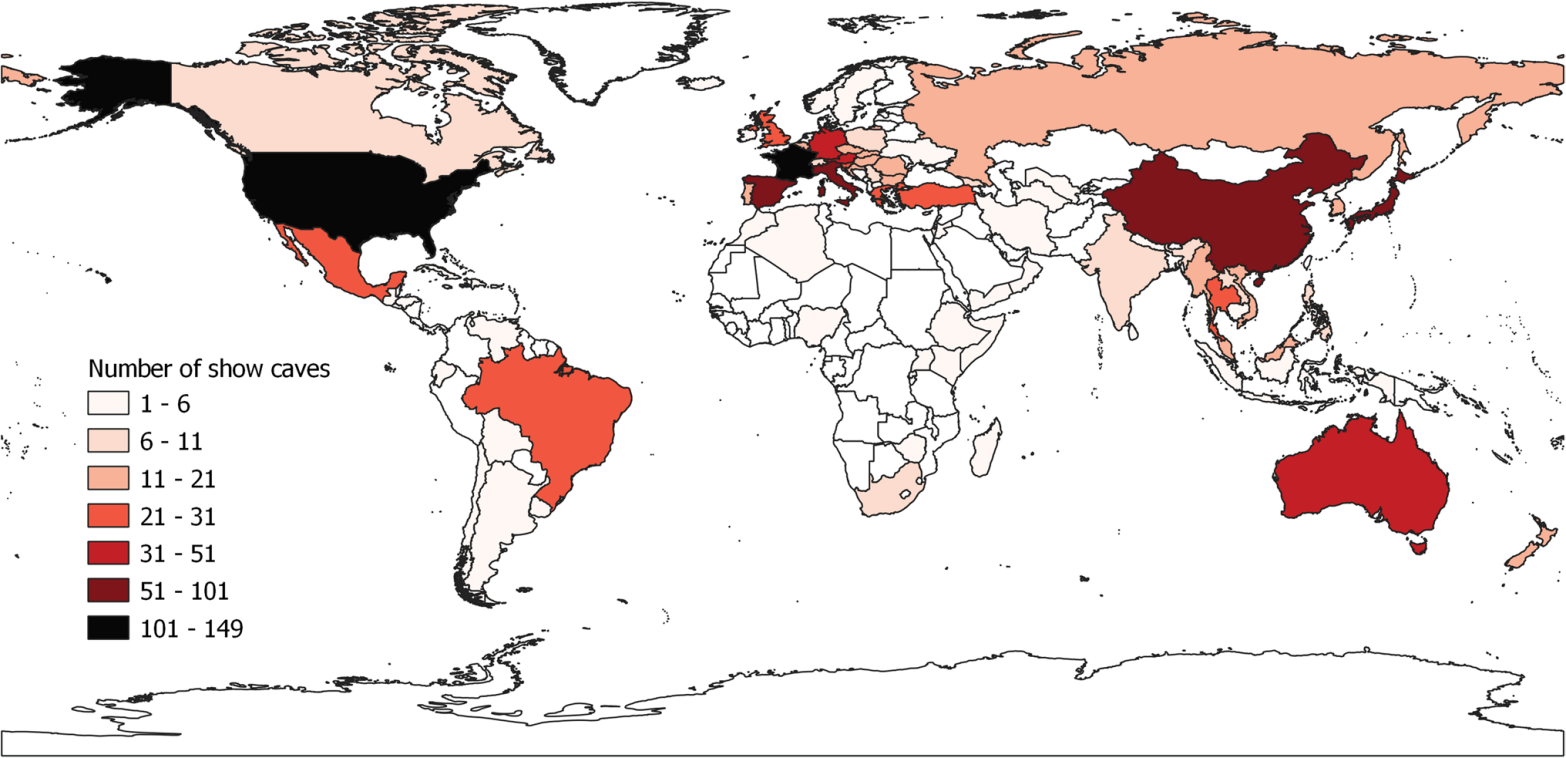
Show cave distribution in the world ordered by country. Darker colour indicates a higher number of show caves

Figure 8 shows the worldwide cave distribution per country. The USA hosts the highest number of show caves (149), followed by France (105) and China (74), while 28 countries out of the total 95 countries have only one show cave in their territory. 45.54% of show caves are found in Europe, followed by Central and Eastern Asia (16.03%) and North America (15.13%); the number of show caves found in South-East Asia, Oceania, South America, Central America, Middle East, and Africa attests between 7.52 and 1.8% (Tab. 2).

**Fig. 9:**
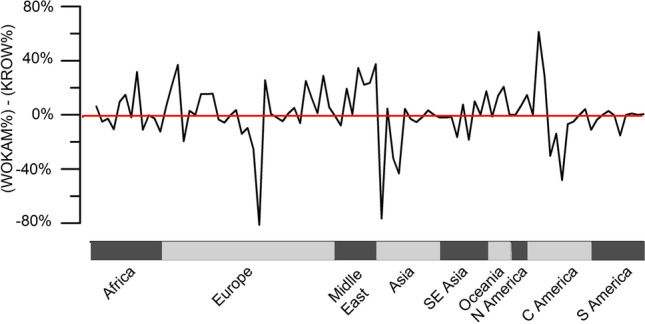
Difference between each country karst percentage calculated using WOKAM and KROW shapefile and subdivided by geographical region

The comparison between the WOKAM and KROW datasets shows some discrepancies (Tab. 2, Fig. 9). The relation between the percentage of karst territories calculated using the two available datasets shows a general overestimation of karst coverage calculated with WOKAM with respect to the ones calculated with KROW dataset (positive difference higher than 30% for: Barbados, Bosnia and Herzegovina, Jordan, and Iran), with few exceptions showing a marked opposite trend and corresponding to data of Monaco, Georgia, Turkmenistan, Uzbekistan, Cuba, and Haiti (Fig. 9). About 40% of the analysed countries presents a difference greater than 10% in calculated karst extension between the two datasets (WOKAM and KROW), but only 11% presents a difference between the two datasets greater than 30%.

The Pearson correlation coefficient between the number of show caves and karst extension in each country with at least one show cave is 0.5 for both datasets (both WOKAM and KROW). At a regional level, the correlation is not significant in Africa, while it is stronger in Asia, Oceania, Central America and South America (Tab. 4). The differences identified using the two datasets suggest that they could be both used for analyses at the global scale, but using them for more detailed and regional investigation would require further research to test their accuracy.

**Table 4:** Pearson correlation coefficients between the number of show caves and karst surface calculated using both the WOKAM and KROW datasets and country GDP (PPP) in each geographical region

Geographical region	R (show cave—WOKAM karst)	R (show cave—KROW karst)	R (show cave—country GDP(PPP)
Africa	-0.2	0.1	0.5
Europe	0.7	0.6	0.1
Middle East	0.1	0.6	0.2
Asia	0.8	0.6	0.5
South-East Asia	-0.3	-0.3	0.6
Oceania	1	1	0.8
North America	0.7	0.3	0.7
Central America	0.8	0.8	-0.2
South America	1	0.9	0
All Regions	0.5	0.5	0.4

**Fig. 10:**
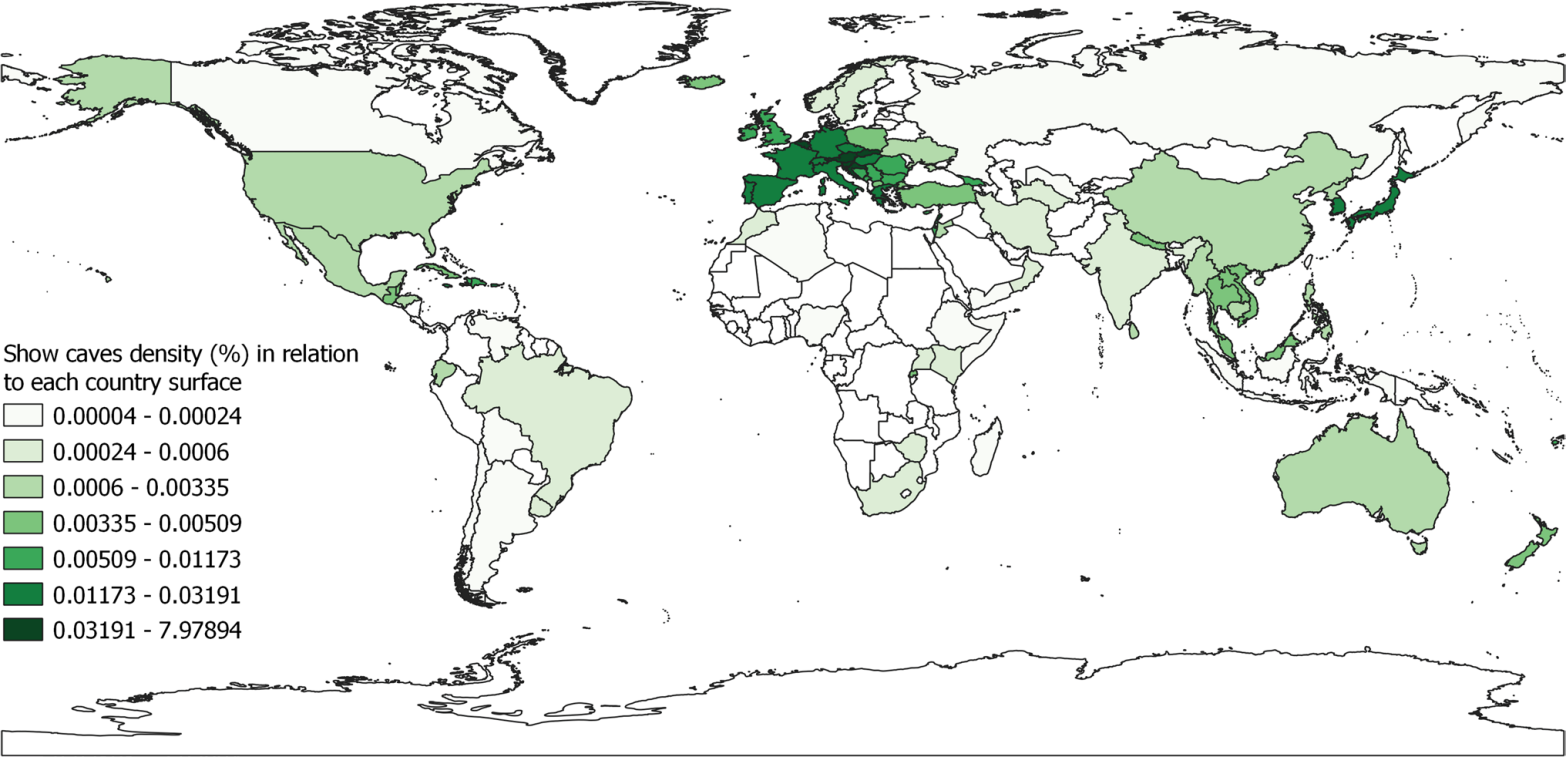
Show cave density (*100) according to each country’s surface area. Darker colour indicates higher density

**Fig. 11:**
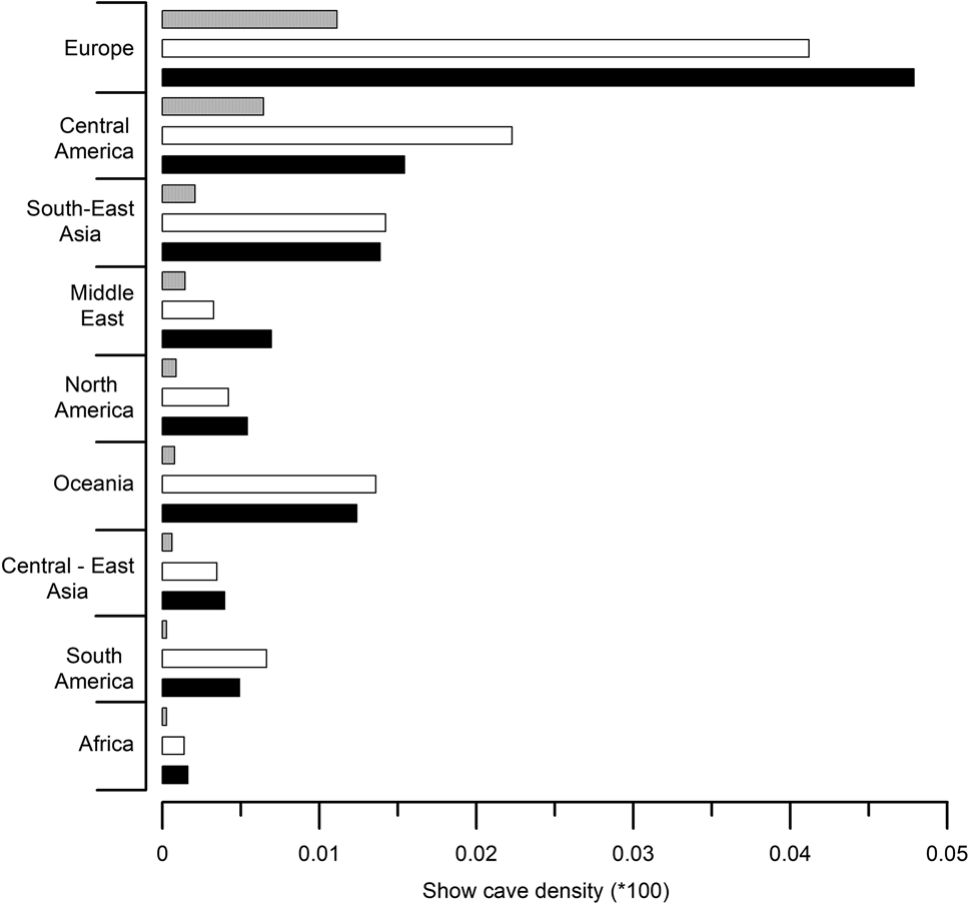
Show cave density in each geographic region according to the surface of countries including at least one show cave (grey bars) and surface of karst areas of the same countries calculated using WOKAM (white bars) and KROW dataset (black bars)

The number of show caves in each country has been compared with both country and karst surface, showing a marked higher density of show caves in Europe (Figs. 10 and 11; Tab. 5). If we exclude islands and small countries where the presence of only one show cave would result in a high density, Slovenia is the country with more show caves according to its territory with a density of 0.002 caves/km^2^. At a regional scale Europe has the highest density, while Africa is the geographical region presenting the lowest density of show caves with respect to country surface (Tab. 5).

**Table 5:** Show cave density (*100) in all countries grouped according to their geographic region

Geographical region	Show cave density/country surface *100	Show cave density/karst WOKAM *100	Show cave density/karst KROW*100
Europe	0.01111	0.04119	0.04790
Central America	0.00644	0.02227	0.01543
South-East Asia	0.00208	0.01423	0.01387
Middle East	0.00144	0.00326	0.00694
North America	0.00087	0.00421	0.00541
Oceania	0.00078	0.013594	0.012387
Central and Eastern Asia	0.00061	0.00347	0.00398
South America	0.00027	0.00663	0.00491
Africa	0.00026	0.00139	0.00162

**Fig. 12:**
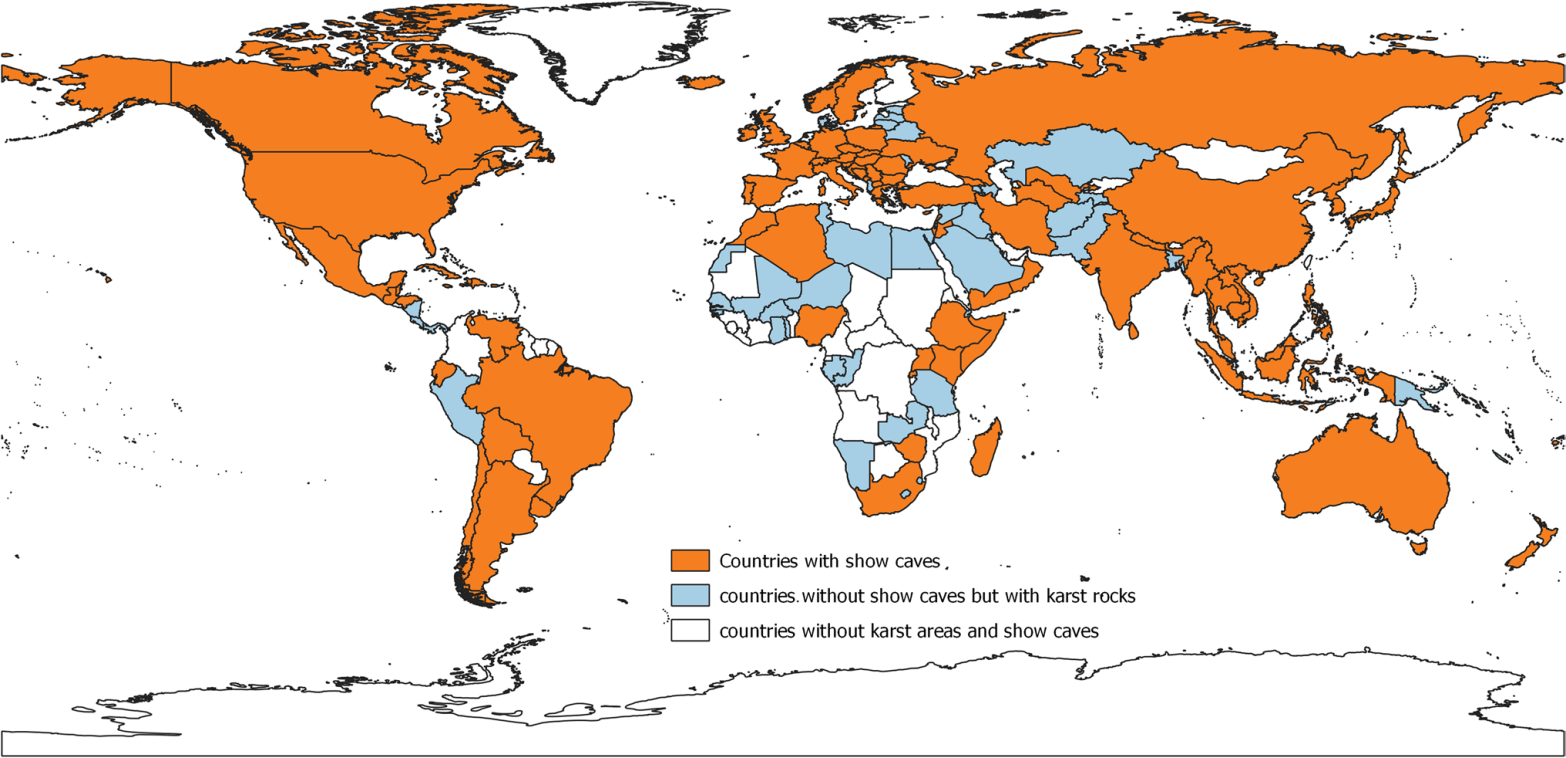
Orange: countries of the world having at least one show cave; blue: countries of the world with a karst coverage higher than 10% of their territory (according to either WOKAM or KROW datasets) but with no known show caves

Considering the relatively low reliability of KROW and WOKAM datasets for high spatial resolution analyses, to identify countries of the world where we have not found important show caves, but having karst rocks in their territory, we decided to select countries with a karst coverage above 10% of their own territory calculated either using KROW and WOKAM datasets (Fig. 12).

**Fig. 13:**
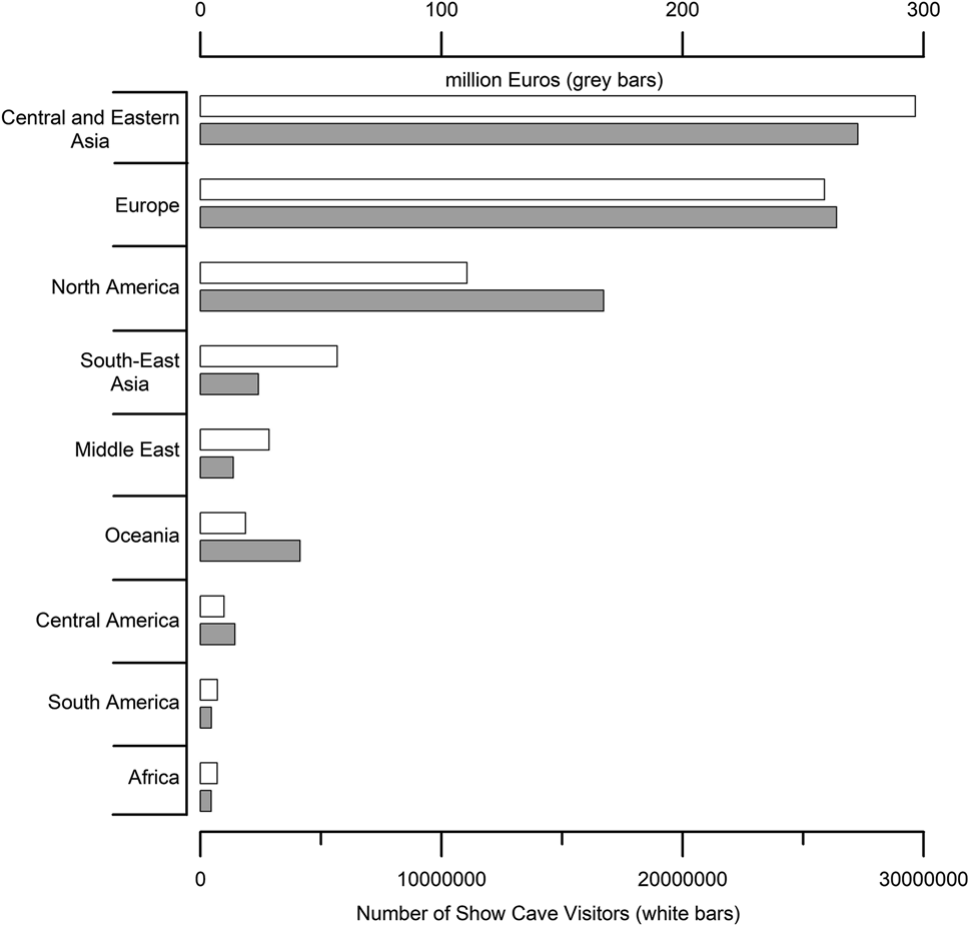
Number of visitors (whited bars) and income from entrance fees (grey bars) of show caves in the chosen geographic regions

The most visited caves can be found in central and eastern Asia, followed by Europe and North America. This also corresponds to the geographical regions where the highest income from show cave entrance fees is registered. Africa, which is the geographic region with the lowest GDP(PPP), is also the one with lowest income and lowest tourism associated with show caves (Fig. 13; Tab. 6).

**Table 6:** Estimated show cave visitors and income in 2019 subdivided by geographic region. The average GDP (PPP)/capita is calculated between the countries with at least one show cave in each geographic region

Geographic region	Number of show caves	Number of visitors (million people)	Total income (million Euros)	Average GDP (PPP)/capita (Euros*10^3^)
Central and Eastern Asia	196	29.66	272.70	14
Europe	557	25.89	263.87	32.03
North America	185	11.06	167.25	34.33
South-East Asia	92	5.68	24.05	9.5
Middle East	41	2.85	13.65	18.3
Oceania	62	1.87	41.29	22
Central America	29	0.99	14.35	16.09
South America	39	0.71	4.54	15
Africa	22	0.70	4.52	5.6

**Fig. 14:**
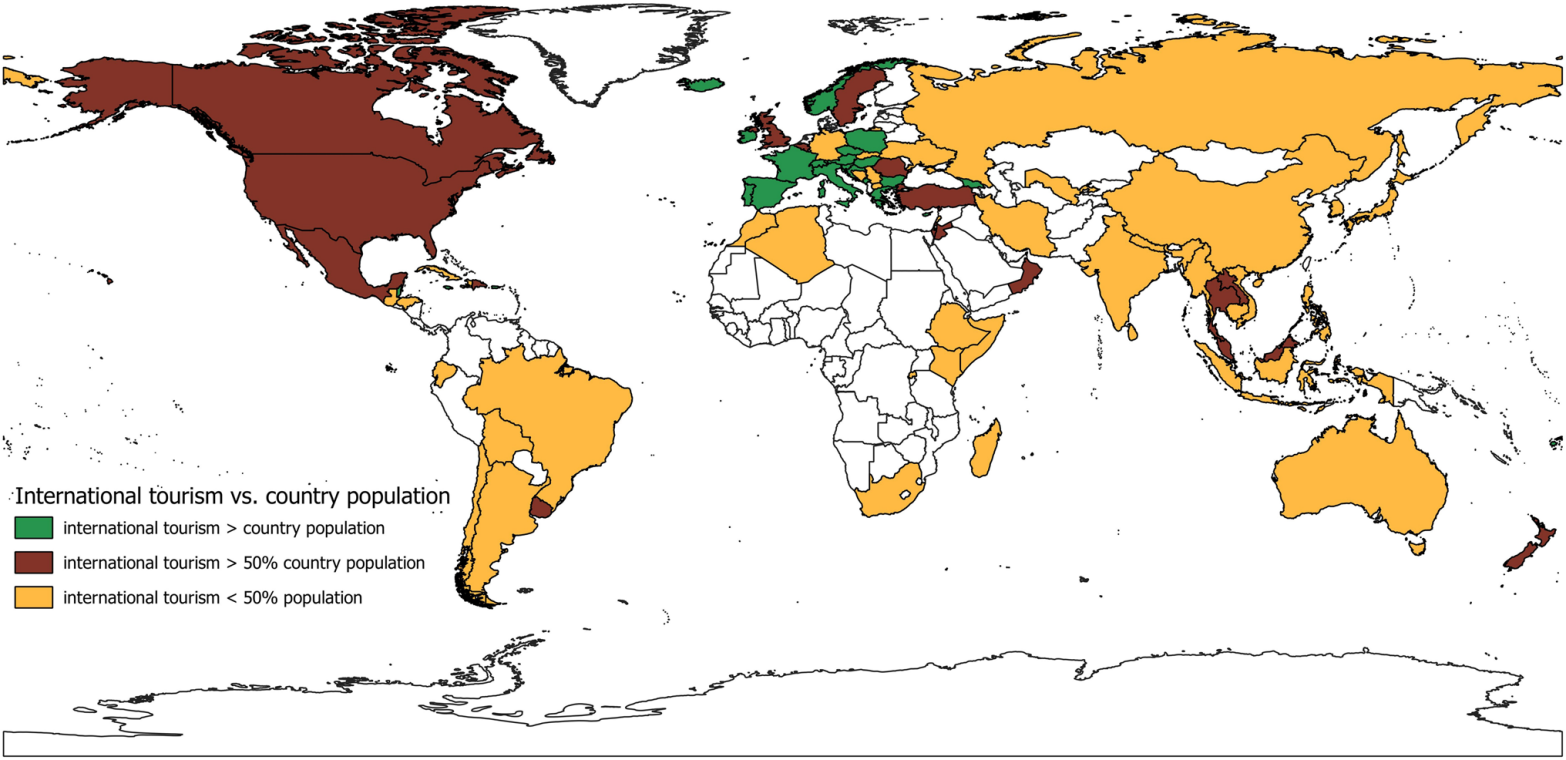
Relation between each country population and international tourists in 2019 in countries with at least one show cave

Finally, to identify the potential importance in show cave economy of international tourism with respect to the domestic one, data about the international presence have been compared with each country population. All countries have been subdivided into three main categories highlighting the ones for which the number of international tourists is greater or lower than 50% of the country’s population, or even exceeds the total population of the country. Although this subdivision does not provide the nationality of show cave visitors, it can give an idea of the wider basin of potential visitors (Fig. 14).

## Present and future of cave tourism

The research carried out using online platforms allowed for the identification of 1223 show caves distributed in 95 countries of the world producing an overall income of almost 800 million Euros for more than 79 million visitors. Although it must be stressed that these data (i.e. total visitors and income) represent an estimation, it is also true that the number of show caves underestimates the actual number of caves open to the public, since all sites not having an official website in English and/or poorly promoted online have been excluded. Thus, the presented data can be considered reliable in providing an order of magnitude for global show cave tourism.

**Fig. 15:**
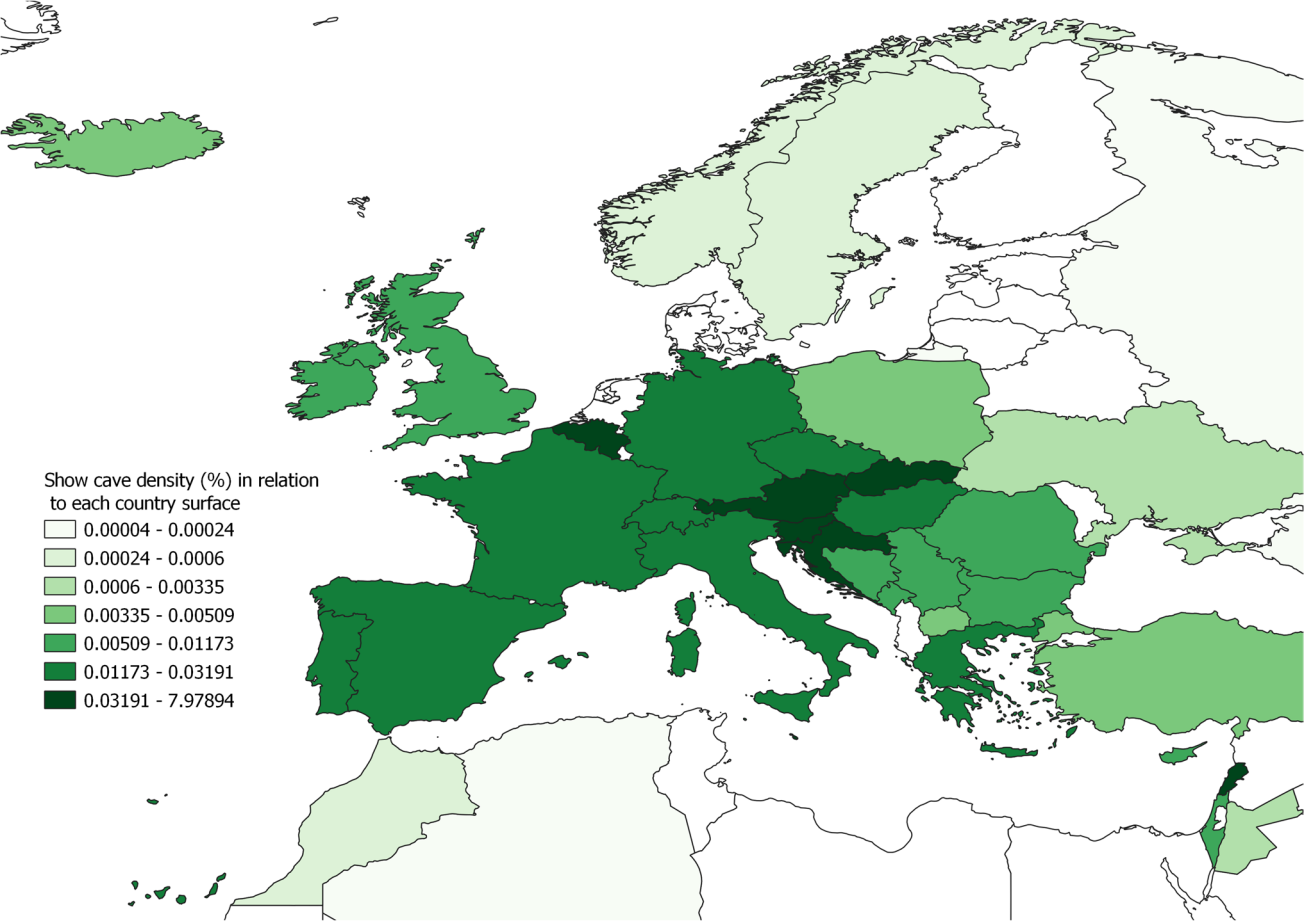
Europe show cave density in relation to each country’s surface area

The distribution of show caves is markedly not uniform with most of them found in the boreal hemisphere (Western countries, China, and Japan). Europe hosts more than 45% of total show caves and has the highest density in relation to its surface (Fig. 10). This distribution clearly reflects the global “maturity” of cave tourism, with Europe showing an older tradition as compared to the rest of the world. Indeed, if we look closer at the European continent, it appears that the highest density of show caves occurs mainly in the northern Balkans, where karst phenomena were first studied, and the first caves have been equipped for tourist visits (Fig. 15).

Considering that the main process leading to cave formation involves the action of water on soluble rocks and the majority of worldwide show caves is found in carbonate rocks (93.71%), the extension of karst formations can be considered a limiting factor for the presence of caves and thus show caves. The analyses carried out using both WOKAM and KROW datasets showed a certain degree of correlation between the number of show caves and karst formations extensions (0.5). Unfortunately, the low resolution of the two datasets at a national scale prevented a deeper data elaboration which might be possible in the future if the resolution of existing datasets will be improved. Indeed, at regional scale the correlation coefficients calculated using both datasets show some discrepancies (Tab. 4), but still indicate that in some regions of the world, the African continent in particular, a correlation between the two variables is lacking. Africa, that is the geographic region with the lowest GDP(PPP), is also the continent hosting the majority of countries without show caves but with relatively vast karst outcrops. Thus, another limiting factor for cave tourism and show cave success must be considered: the country’s economic development and the presence of a well-established tourism network.

Although Europe hosts the majority of show caves, the most visited ones are found in central and eastern Asia (especially in China). Interestingly, in this region international tourism is relatively low if compared to local population, while Europe, which holds the second place in the ranking of most visited show caves, presents opposite conditions (Fig. 14), thus suggesting a potential prominent importance of domestic tourism in Chinese show caves which may be further enlarged to international visitors. The number of show caves has markedly increased in the last decades from the 500 “major” show caves cited by Cigna and Forti (2013) to the 1223 identified in this work, and considering the important income produced by this subterranean tourism, a further increase is expected in the near future. However, although caves may be considered natural resources that can be exploited to improve local economy, they are extremely fragile environments hosting an enormous scientific and, in some situations, archaeological heritage which can be permanently damaged by mass tourism or by an incorrect show cave management. Conversely, show caves can also be an excellent tool for cave conservation. If the cave is chosen carefully, the existing guidelines are strictly followed, cave guides are specifically trained, and best management practices are maintained through time, then opening a cave to tourists can protect the cave itself from uncontrolled visitors while becoming an excellent opportunity for environmental education and scientific advertising among the wide public. As an example, many show caves have installed temporary or permanent exhibits regarding caves and karst, speleology, or peculiar scientific interests (Columbu et al., 2021). Another aspect that must never be forgotten is the economic feasibility. Opening a show cave requires not only a detailed scientific investigation, but also a specific market survey addressed to the future economic sustainability of the show cave. When equipping a cave for tourist visits some permanent damages will always be introduced, thus if the cave is abandoned soon after its opening because of low tourist affluence, then a double damage is introduced: the first one produced within the show cave development phase and the second related to the abandonment of an easily accessible cave which will be left at the mercy of uncontrolled visitors.

## Conclusions

A detailed research of global show caves has been carried out within this study, leading to the compilation of a database of worldwide show caves including some geologic (hosting bedrock) and economic (number of visitors, entrance fees, etc.) information, associated with a summary of existing guidelines for sustainable show cave development and management.

A global picture of current show cave tourism has been produced showing a prominent presence of show caves in Europe, followed by Western countries (USA and Australia), China, and Japan.

A total number of 1223 show caves visited by more than 79 million people and providing almost 800 million Euros income (entrance fees only) witnesses the high potential of these natural sites in the tourism industry and the potential further development of show caves. In addition, the increasing demand for adventure tourism may boost the number of speleological guided tours, making it necessary to develop specific guidelines to regulate this high-impact activity. Caves are fragile but extremely important environments which can be permanently damaged by irresponsible tourism. On the other hand, opening a show cave can be an excellent tool for the protection of the cave and karst environment and scientific promotion. Following the international guidelines for show cave development such as the ISCA, UIS, and IUCN guidelines, carrying out a continuous monitoring of cave parameters and investing in cave guide training would ensure not only the conservation of these delicate and rich geo-ecosystem, but also of the beauties attracting tourists, thus preserving also the economic value of these environments.

## Supplementary Information

Below is the link to the electronic supplementary material.

Supplementary file1 (XLSX 92 KB)

Supplementary file2 (DOCX 13 KB)

Supplementary file3 (CPG 0 KB)

Supplementary file4 (DBF 703 KB)

Supplementary file5 (PRJ 0 KB)

Supplementary file6 (QMD 1 KB)

Supplementary file7 (SHP 43 KB)

Supplementary file8 (SHX 10 KB)
