# Supplementary material for: A Global Perspective on Sustainable Show Cave Tourism
Source: Geoheritage. 2022 Jun 28;14(3):82. doi: 10.1007/s12371-022-00717-5 (PMC9244007; doi:10.1007/s12371-022-00717-5)
Supplement: Supplementary file 2 — Supplementary file2 (DOCX 13 KB) [file 12371_2022_717_MOESM2_ESM.docx]

Article Title: A global perspective on sustainable show cave tourism

Journal Name: Geoheritage

Authors: Veronica Chiarini ^1^(*), Jochen Duckeck ^2^, Jo De Waele ^1^

Affiliations: ^1^ Department of Biological, Geological and Environmental Sciences, University of Bologna, Via Zamboni 67, 40126 Bologna, Italy. E-mail: veronica.chiarini3@gmail.com; [jo.dewaele@unibo.it](mailto:jo.dewaele@unibo.it);

^2^ Archivstr. 11, 90408 Nürnberg, Germany. E-mail: [octavian@showcaves.com](mailto:octavian@showcaves.com).

(*) corresponding author

Caption: **Online Resource 2** Show caves of the world georeferenced

File description: Shapefile with all show caves listed in online_resource1 georeferenced. The attribute table include each show cave name, the country where it is found and type of bedrock.
